# Membrane protein Bcsdr2 mediates biofilm integrity, hyphal growth and virulence of *Botrytis cinerea*

**DOI:** 10.1007/s00253-024-13238-8

**Published:** 2024-06-28

**Authors:** Wei Zhang, Yi Cao, Hua Li, Abdel-Hamied M. Rasmey, Kecheng Zhang, Liming Shi, Beibei Ge

**Affiliations:** 1 State Key Laboratory of Biology of Plant Diseases and Insect Pests, Institute of Plant Protection, Chinese Academy of Agricultural Sciences, 2 Yuanmingyuan West Road, Beijing, 100193 China; 2 Qian Xinan Branch of Guizhou Provincial Tobacco Company, 60 Ruijin Southern Road, Xingyi, 562499 China; 3 Guizhou Academy of Tobacco Science, 29 Longtanba Road, Guiyang, 550081 China; 4 School of Light Industry Science and Engineering, Beijing Technology and Business University, 11 & 33 Fucheng Road, Beijing, 100048 China; 5 Botany and Microbiology Department, Faculty of Science, Suez University, Elsalam 1, Cairo-Suez Road, Suez, 43221 Egypt

**Keywords:** *Botrytis cinerea*, Bcsdr2, Hyphal growth, Stress tolerance, Biofilm integrity, Pathogenicity

## Abstract

**Abstract:**

Grey mould caused by *Botrytis cinerea* is a devastating disease responsible for large losses to agricultural production, and *B. cinerea* is a necrotrophic model fungal plant pathogen. Membrane proteins are important targets of fungicides and hotspots in the research and development of fungicide products. Wuyiencin affects the permeability and pathogenicity of *B. cinerea*, parallel reaction monitoring revealed the association of membrane protein Bcsdr2, and the bacteriostatic mechanism of wuyiencin was elucidated. In the present work, we generated and characterised Δ*Bcsdr2* deletion and complemented mutant *B. cinerea* strains. The Δ*Bcsdr2* deletion mutants exhibited biofilm loss and dissolution, and their functional activity was illustrated by reduced necrotic colonisation on strawberry and grape fruits. Targeted deletion of Bcsdr2 also blocked several phenotypic defects in aspects of mycelial growth, conidiation and virulence. All phenotypic defects were restored by targeted gene complementation. The roles of Bcsdr2 in biofilms and pathogenicity were also supported by quantitative real-time RT-PCR results showing that phosphatidylserine decarboxylase synthesis gene *Bcpsd* and chitin synthase gene *BcCHSV* *II* were downregulated in the early stages of infection for the Δ*Bcsdr2* strain. The results suggest that Bcsdr2 plays important roles in regulating various cellular processes in *B. cinerea*.

**Key points:**

*• The mechanism of wuyiencin inhibits B. cinerea is closely associated with membrane proteins.*

*• Wuyiencin can downregulate the expression of the membrane protein Bcsdr2 in B. cinerea.*

*• Bcsdr2 is involved in regulating B. cinerea virulence, growth and development.*

**Supplementary Information:**

The online version contains supplementary material available at 10.1007/s00253-024-13238-8.

## Introduction

*Botrytis cinerea* Pers.: Fr. (teleomorph: *Botryotinia fuckeliana* Whetzel) is a typical necrotrophic ascomycete and worldwide plant pathogen that infects crop hosts during both pre- and post-harvesting phases (Bu et al. 2021; Fan et al. 2023). Moreover, *B. cinerea* infects > 1400 plant species including many economically important crops, leading to tremendous economic losses (Elad 2016). Due to the lack of resistant varieties, chemical control remains the most effective strategy for managing grey mould caused by *B. cinerea* (Weidensdorfer et al. 2019). However, through genetic plasticity, *B. cinerea* has developed resistance to many types of fungicides (Liu et al. 2021). Researchers use *B. cinerea* as a model fungus in molecular studies, therefore, exploring the molecular mechanisms underlying the development and virulence of *B. cinerea* will contribute to establishing more effective disease control strategies.

Membrane proteins play key roles in the physiological processes of microorganisms including transportation and intercellular communication, and they serve as important drug targets. Membrane proteins are recognised and inserted into the lipid bilayer by exquisite cellular machineries such as GlpG rhomboid protease, which is thought to allow the docking of a transmembrane substrate (Engberg et al. 2022). Transporters are integral membrane proteins with central roles in the efficient movement of molecules across biological membranes. The nucleobase ascorbate transporter UapA from *Aspergillus nidulans* must dimerise for correct trafficking to the membrane (Alguel et al. 2016). Many membrane proteins are primary drug targets, especially those involved in converting extracellular signals into intracellular processes. Among them, G protein-coupled receptors (GPCRs) are crucial for cellular responses to a range of bioactive molecules, and they play a key role in signalling, such as increasing the basal activity of the cannabinoid 2 receptor (Yeliseev et al. 2021). Interestingly, cell membrane proteins are important targets of fungicides in the prevention and control of fungal diseases (Huang et al. 2022). For example, natamycin inhibits the growth of yeasts and other fungi by inhibiting plasma membrane transporters that regulate amino acid and glucose transport (te Welscher et al. 2012).

Wuyiencin is a nucleoside biological fungicide that has a significant control effect on *B. cinerea*. Previous analysis in our laboratory found that wuyiencin can inhibit *B. cinerea* by affecting mycelial morphology and intracellular structure (Sun et al. 2003). Using proteomics, we identified 27 proteins that are significantly regulated by wuyiencin in *B. cinerea* (Shi et al. 2019). Cell membrane proteins mediate the effects of fungicides in the prevention and control of fungal diseases by fungicides (Huang et al. 2022). Since wuyiencin can inhibit protein synthesis and increase cell membrane permeability of *B. cinerea*, we highlighted the downregulated membrane protein Bcsdr2 in network-based comparative genomic analysis, but the functions of Bcsdr2 remain poorly understood. Through homologous protein searches, we found that homologs of Bcsdr2 included alcohol dehydrogenase (BcADH1), short-chain dehydrogenase/reductase (YdfG and YMR226C), NADPH-dependent 1-acyldihydroxyacetone phosphate reductase (AYR1) and others. Among them, BcADH1 can mediate spore germination, hyphal growth and sclerotia formation in *B. cinerea*, decrease sporulation and affect pathogenicity and oxidative stress ability (DafaAlla et al. 2022). YdfG and YMR226C are NADP( +)-dependent short-chain dehydrogenases with L-serine dehydrogenase activity, and they play important roles in maintaining normal physiological metabolism in *Escherichia coli* and *Saccharomyces cerevisiae* (Fujisawa et al. 2003). Deletion of Ayr1 results in the loss of 1-acylglycerol phosphate reductase activity, which inhibited the synthesis of *S. cerevisiae* lipids and spore germination (Athenstaedt and Daum 2000). In the present study, we investigated the role of membrane protein Bcsdr2 in fungal growth and pathogenesis to reveal and explore the role of wuyiencin in controlling *B. cinerea*.

## Materials and methods

### Fungal strains and culture conditions

Strain B05.10 of *B. cinerea* Pers.: Fr. (*B. fuckeliana* (de Bary) Whetzel) was originally isolated from *Vitis vinifera* and has been widely used as a standard reference strain (Quidde et al. 1999). The strain B05.10 of *B. cinerea* was gifted by Associate Professor Liu Pengfei of China Agricultural University. *B. cinerea* was grown on potato dextrose agar (PDA; 200 g potato, 20 g dextrose, 20 g agar, 1 L water), complete medium (CM; 1 g yeast extract, 0.5 g casein acid hydrolysate, 0.5 g hydrolysed casein, 10 g glucose, 4 mM Ca(NO_3_)_2_ꞏ4H_2_O, 1.5 mM KH_2_PO_4_, 1 mM MgSO_4_·7H_2_O, 2.5 mM NaCl, 20 g agar, 1 L water) and minimal medium (MM; 10 mM K_2_HPO_4_, 10 mM KH_2_PO_4_, 4 mM (NH_4_)_2_SO_4_, 2.5 mM NaCl, 2 mM MgSO_4_, 0.45 mM CaCl_2_, 9 μM FeSO_4_, 10 mM glucose, 20 g agar, 1 L water, pH 6.9).

Conidia were quantified after 10 days of incubation on PDA medium, washed from plates, diluted to 5 mL with ddH_2_O and counted under a microscope. Growth tests under different stress conditions were performed on PDA plates supplemented with different agents including H_2_O_2_, KCl and sodium dodecyl sulphate (SDS) (Yan et al. 2010). The percentage of mycelial radial growth inhibition (RGI) was calculated using the formula RGI = ([C – N] / [C − 5]) × 100, where *C* and *N* indicate the colony diameter of control and treatments, respectively (Yang et al. 2018). Each experiment was repeated three times.

### Gene deletion and complementation

To replace the *Bcsdr2* gene in wild type (WT) B05.10 strain, 1000 bp upstream and 1000 bp downstream flanking sequences of *Bcsdr2* were amplified by PCR from genomic DNA of B05.10. The resulting amplicons were fused with the *HPH* (hygromycin resistance gene) using double-joint PCR (Yu et al. 2004). Protoplast preparation and transformation were performed as previously described (Gronover et al. 2001). The resulting hygromycin-resistant transformants were preliminarily screened by PCR with primers (Supplementary Table S1) and further confirmed by Southern blotting analysis. The hygromycin B resistance fragment was used as a probe and labelled with digoxigenin (DIG) using a High Prime DNA Labelling and Detection Starter Kit I (Roche Diagnostics, Mannheim, Germany) according to the manufacturer’s protocol. Genomic DNA was digested with *Eco*RI endonuclease. For complementation assays, a *Bcsdr2*-green fluorescent protein (GFP) cassette was generated by amplifying the entire open reading frame (ORF) of *Bcsdr2* (without a stop codon) and cloning into the pNAN-OGG vector containing the *GFP* gene and the nourseothricin resistance gene (Ren et al. 2018). The resulting construct was confirmed by DNA sequencing and transformed into the *Bcsdr2* deletion mutant.

### Transcriptome analyses

Mycelia of WT B05.10 and *Bcsdr2* gene deletion mutant Δ*Bcsdr2* strains were harvested after growth on PDA medium at 22 °C under 12-h light and 12-h dark conditions for 3 days (with three biological replicates). Total RNA was extracted using a fungal RNA kit(R6840-01; Omega Bio-Tek, Norcross, GA, USA) and tested for quality using Nanodrop. RNA sample having a RNA integrity number > 7.0, 260/280 ratio > 1.8 and 260/230 ratio > 1.9 were analysed by Allwegene Technology Co., Ltd. (Beijing, China). Briefly, Trimmomatic (v0.33) software was used to filter the sequencing data (Goulin et al. 2023). A reference genome index was built, and filtered reads were mapped to the reference genome using STAR (v2.5.2b) (Acevedo et al. 2024). Mapping statistics are shown in Supplementary Table S2. HTSeq (v0.5.4) was used to compare the read count values for each gene with the original gene expression level, and fragments per kilobase of exon model per million mapped reads (FPKM) were used to standardise expression. DESeq (v1.10.1) was used to analyse differentially expressed genes (DEGs) with absolute log2 value > 1 and *p* < 0.05 as cut-off criteria (Saputro et al. 2023). All DEGs are listed in Supplementary Table S3. Gene Ontology (GO) categories of up- and downregulated genes were identified using the g:Profiler toolset (Raudvere et al. 2019).

### Nucleic acid manipulation and qRT-PCR

Fungal genomic DNA was extracted as described previously by McDonald and Martinez (1990). Plasmid DNA was isolated using a RapidLyse Plasmid Mini Kit (DC211; Vazyme, Nanjing, China).

Real-time quantitative reverse transcription (qRT-PCR) was used to measure the expression of disease-related genes in the *Bcsdr2* disruption mutant Δ*Bcsdr2* and the WT B05.10 strain. The total RNA remaining from the transcriptome experiment was used. RNA was reverse-transcribed using a HiScript III 1st Strand cDNA Synthesis Kit (R312; Vazyme). qRT-PCR was performed using Taq Pro Universal SYBR qPCR Master Mix (Q712; Vazyme). The expression level of each transcript was calculated using the ΔΔC_t_ method (Livak and Schmittgen 2001). For normalisation of the data, the transcription level of each gene in hyphae of strain B05.10 was given a value of 1.0, and the scale was used to calibrate the transcript levels of genes in hyphae of Δ*Bcsdr2*. qRT-PCR was repeated three times. All genes and primers used for qRT-PCR are listed in Supplementary Table S4.

### Pathogenicity and infection-related morphogenesis assay

Infection tests were performed on grape fruits and leaves. Briefly, the tested plant tissues were point-inoculated with 5-mm-diameter mycelial plugs of 3-day-old cultures. Before inoculation, the cuticle of the hosts was wounded with a sterilised needle tip to facilitate penetration of the fungus into plant tissues. The inoculated samples were placed under high relative humidity conditions (~ 95%) at 25 °C with 16 h of daylight. These experiments were repeated three times, and each included ten samples. Infection-related morphogenesis was observed on the onion epidermis using a published method (Zhang et al. 2022).

### Morphology and ultrastructure of fungal hyphae

To investigate the role of Bcsdr2 on hyphal morphology and ultrastructure in *∆Bcsdr2* and WT B05.10 strains, scanning electron microscopy (SEM) and transmission electron microscopy (TEM) were performed. Mycelial morphology and ultrastructure were observed by SEM/TEM according to a modified method (Zou et al. 2022). Hyphae on coverslips were immersed in 4 °C glutaraldehyde (4%) and incubated in darkness at 4 °C for 16 h. Mycelia were washed three times with phosphate-buffered saline (PBS), dehydrated and dried in a vacuum freeze-dryer. Samples were sprayed with gold powder and examined using an SU8000 SEM instrument (Hitachi, Tokyo, Japan). One millilitre of spore suspension (1 × 10^5^ spores mL^−1^) was added to 100 mL of PDB and incubated at 25 °C with shaking a 150 rpm for 72 h. Mycelia were centrifuged, washed three times with PBS and postfixed with 1% osmium tetroxide for 2 h. Samples were washed three more times with PBS, further dehydrated in a graded ethanol series (30%, 50%, 60%, 70%, 80%, 90%, 95% and 100%) and then embedded in Spurr’s low viscosity resin. Sections were observed using an H-7500 TEM instrument (Hitachi, Tokyo, Japan).

### Abiotic stress and pathogenic factor assay

Mycelia-responsive trials were carried out to determine the responses of B05.10, Δ*Bcsdr2* and complemented strain Δ*Bcsdr2*-C to abiotic stresses, including osmotic pressure, H_2_O_2_, sodium dodecyl sulphate (SDS), protease, polygalacturonase and cellulase, and their ability to produce infectious agent. Specifically, mycelial agar plugs were removed from the margin area of a 2-day-old PDA culture of an isolate and inoculated in Petri dishes containing PDA with KCl (1 M), H_2_O_2_ (10 mM) and 20 mg/L SDS (w/v). Cultures were incubated at 20 °C for 2 days. Secretion of proteases, polygalacturonases and cellulases was assessed using nutrient agar (NA; 5 g NaCl, 10 g sucrose, 3 g beef extract, 3 g yeast extract, 20 g agar, 1 L water, pH 7.0), polygalacuronic acid agar (PGAA; 10 g polygalacuronic acid, 20 g sucrose, 2 g (NH_4_)_2_SO_4_, 20 g agar, 1 L water) and carboxymethyl cellulose sodium agar (CMC-Na; 10 g carboxymethyl cellulose sodium salt, 10 g yeast extract, 1 g tryptone, 4 g (NH_4_)_2_SO_4_, 2 g K_2_HPO_4_, 0.5 g MgSO_4_ꞏ7H_2_O, 20 g agar, 1 L water) medium, respectively. Cultures were incubated at 22 °C for 3 days. Experiments included one mycelial agar plug per dish and three dishes (replicates) for each treatment. The diameter of each colony was measured, and the mycelial growth inhibition rate (MGIR) was calculated using the following formula (Zhou et al. 2022):

$$\text{MGIR}=\left(\text{ADCK}-\text{Ds}\right)/\text{ADCK}\times 100\text{\%}$$

where ADCK is the average colony diameter of an investigated isolate in the control treatment, and Ds is the diameter of that isolate in the presence of a stress generation chemical (KCl, H_2_O_2_ or SDS). Each assay was repeated three times.

### Statistical analyses

All assays were conducted in triplicate unless otherwise indicated. The number of conidia, colony diameter and lesion diameter date was assessed using IBM SPSS statistics 20.0 software (IBM Corp., Armonk, NY, USA). The significance of different treatments on various indices was evaluated by analysis of variance (ANOVA) with least significant difference (LSD’s) multiple comparisons, and *p* ≤ 0.05 was considered statistically significant. After analysis, the average angular values were individually back-transformed to numerical values.

## Results

### Identification of Bcsdr2 in *B. cinerea*

**Fig. 1:**
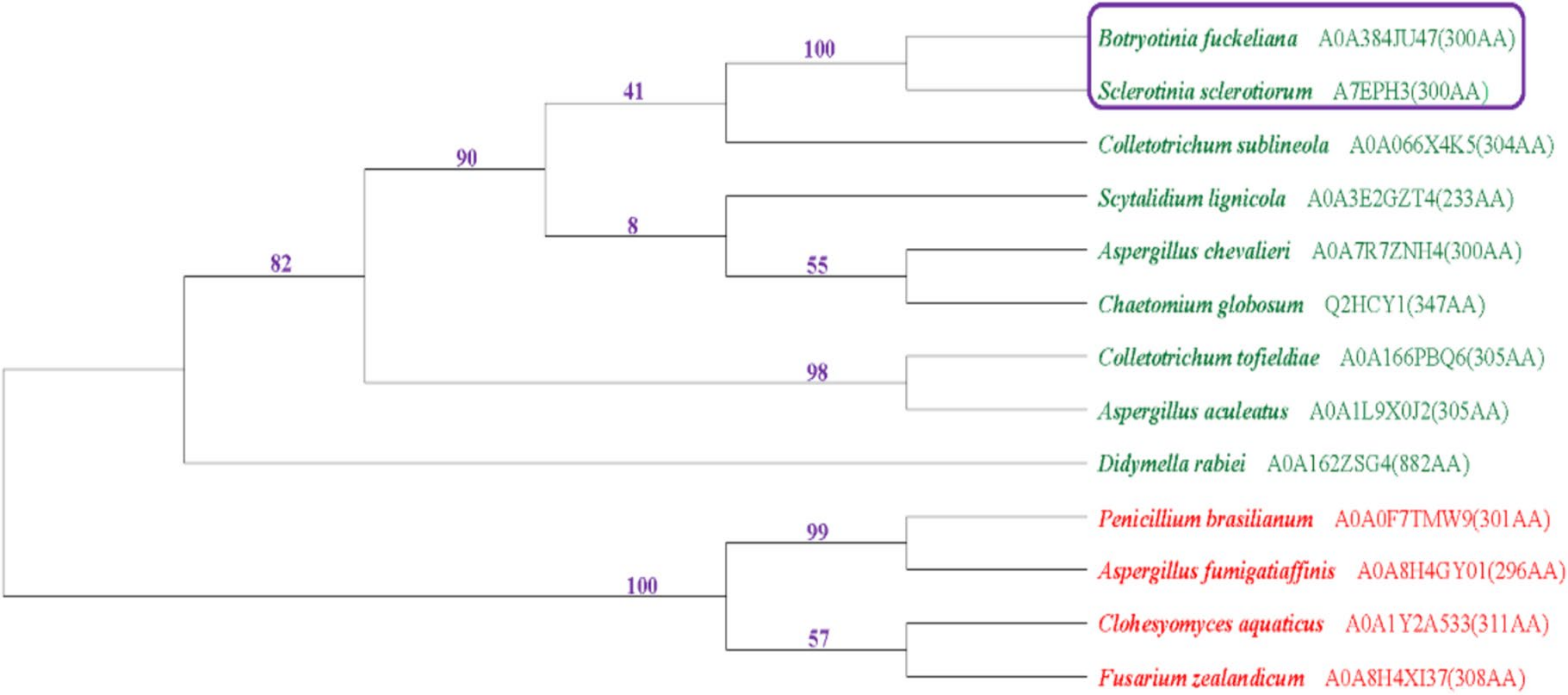
Phylogenetic tree of Bcsdr2 proteins based on a neighbour-joining analysis using MEGA. Numbers represent the boostrap values

The *Bcsdr2* gene (Bcin10g01350) of *B. cinerea* was identified by transcriptome data analysis. Bioinformatics analysis by NCBI (https://www.ncbi.nlm.nih.gov/) showed that this 1505-bp gene comprising three exons and two introns encodes 300 amino acid polypeptide. Homologs of Bcsdr2 were identified by BLASTp and phylogenetic trees of Bcsdr2 proteins were constructed by MEGA 10.0.5 (Fig. 1) (Tribhuvan et al. 2022). The evolutionary history was inferred using the neighbour-joining method with bootstrap replications 1000 (Russo and Selvatti 2018). The Bcsdr2 protein is highly homologous to EMR85959.1 and P024480.1 from *B. cinerea* BcDW1 and Bofu T4 strains, respectively.

### Deletion and complementation of Bcsdr2 in *B. cinerea*

**Fig. 2:**
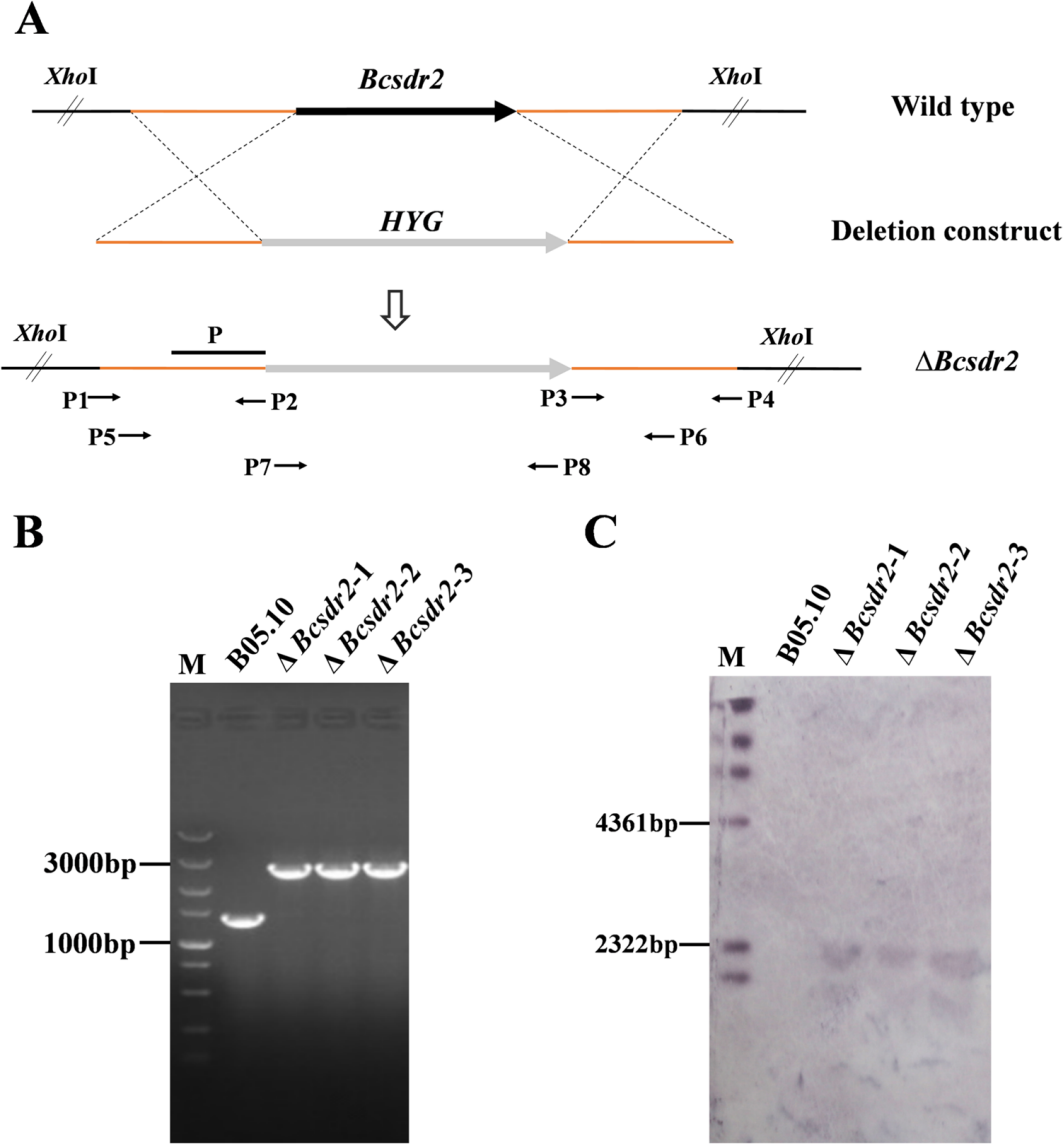
Target gene deletion. **A** Schematic diagram of the *Bcsdr2* homologous replacement strategy. **B** Amplification of *Bcsdr2* recombinant fragments in B05.10 and *Bcsdr2* gene deletion mutants. **C** Southern blotting of *Bcsdr2* gene deletion mutant strains

To investigate the functions of the Bcsdr2 protein in *B. cinerea*, we generated single Δ*Bcsdr2* gene deletion mutants using homologous recombination (Fig. 2A). The left and right arms (1000 bp) of the *Bcsdr2* gene and the hygromycin B resistance gene (2145 bp) of plasmid pUCHYG were amplified, and the recombinant *Bcsdr2* gene containing the above fragments was obtained by fusion PCR.

We obtained independent transformants by screening on selection medium supplemented with hygromycin B and PCR verification. After single spore isolation, transformants were verified as homozygous by PCR and further confirmed to be single-copy insertions by Southern blotting analysis (Fig. 2 B and C). To confirm that the phenotypic changes of mutants were due to gene deletion, Δ*Bcsdr2* mutants were complemented with the full-length *Bcsdr2* gene to generate Δ*Bcsdr2*-C complemented strains.

### Bcsdr2 is involved in hyphal growth and conidiation

**Fig. 3:**
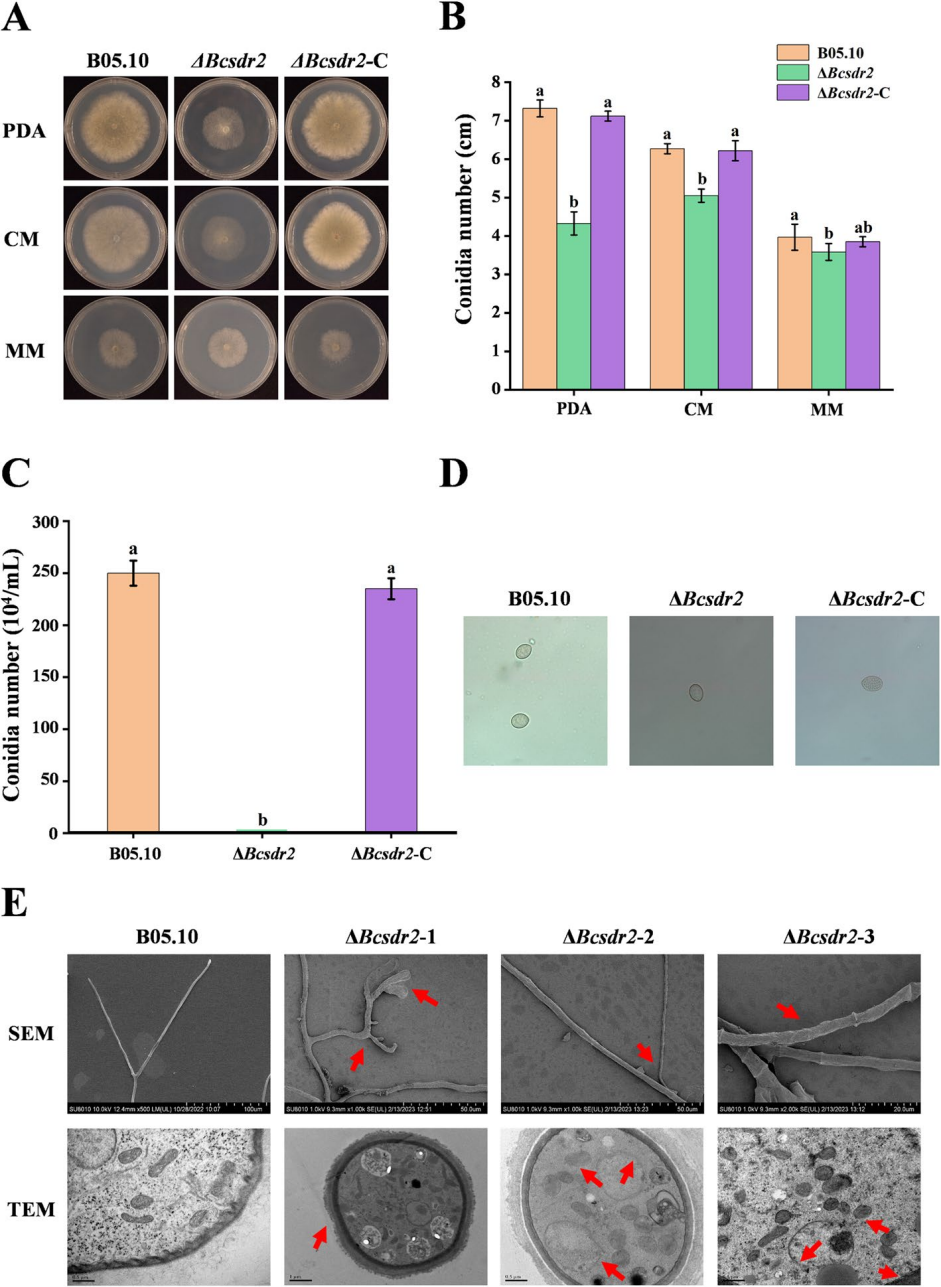
Effects of Bcsdr2 deletion on mycelial growth, sporulation and conidial germination. Bars represent standard errors from three replicates. Values on bars followed by different letters indicate significant differences at *p* = 0.05. **A** Mycelial growth of *∆Bcsdr2*, B05.10 and *∆Bcsdr2*-C strains on PDA plates after 3 days of cultivation. **B** Quantification of colony diameter of the indicated strains grown on PDA plates for 3 days. **C** Quantification of conidia produced by the indicated strains. **D** Conidia morphology of different strains. **E** SEM and TEM observations of hyphae of *B. cinerea* and *∆Bcsdr2* strains grown on PDA plates (diameter 4 cm)

The mycelial growth rate and conidia of Δ*Bcsdr2* were significantly different from the WT parent B05.10. The Δ*Bcsdr2* strain had a slower growth rate than the Δ*Bcsdr2*-C complemented strains and the B05.10 WT strain on PDA, CM and MM, especially on PDA (Fig. 3 A and B). Conidia are the primary inoculum for the disease cycle of *B. cinerea* (Abbey et al. 2019). After incubating on PDA for 10 days, the abundance of conidia was significantly less for Δ*Bcsdr2* than for B05.10 and Δ*Bcsdr2*-C (Fig. 3C). However, there were no significant changes in morphology or size (Fig. 3D). In addition, when incubated on PDA medium at 22 °C for 10 h, all spores of B05.10 germinated, whereas the average germination rate of Δ*Bcsdr2* was only 75.32%.

SEM results showed that hyphae of control strain B05.10 grew upright, with uniform thickness, a smooth surface and no distortion or deformities, and apical hyphae branches were relatively uniform; by contrast, those of Δ*Bcsdr2* displayed swollen tips, reduced hyphal size and uneven apical branches. TEM results showed that compared with control strain B05.10, the Δ*Bcsdr2* strain exhibited dissolution and disappearance of various biological membranes, including mitochondrial membranes, endoplasmic reticulum membranes, nuclear membranes and cell membranes. The above features are indicated by the red arrows in the figure (Fig. 3E). These results also indicate that *Bcsdr2* is important for vegetative growth and conidiation of *B. cinerea*.

### Bcsdr2 participates in regulating the pathogenicity of *B. cinerea*

**Fig. 4:**
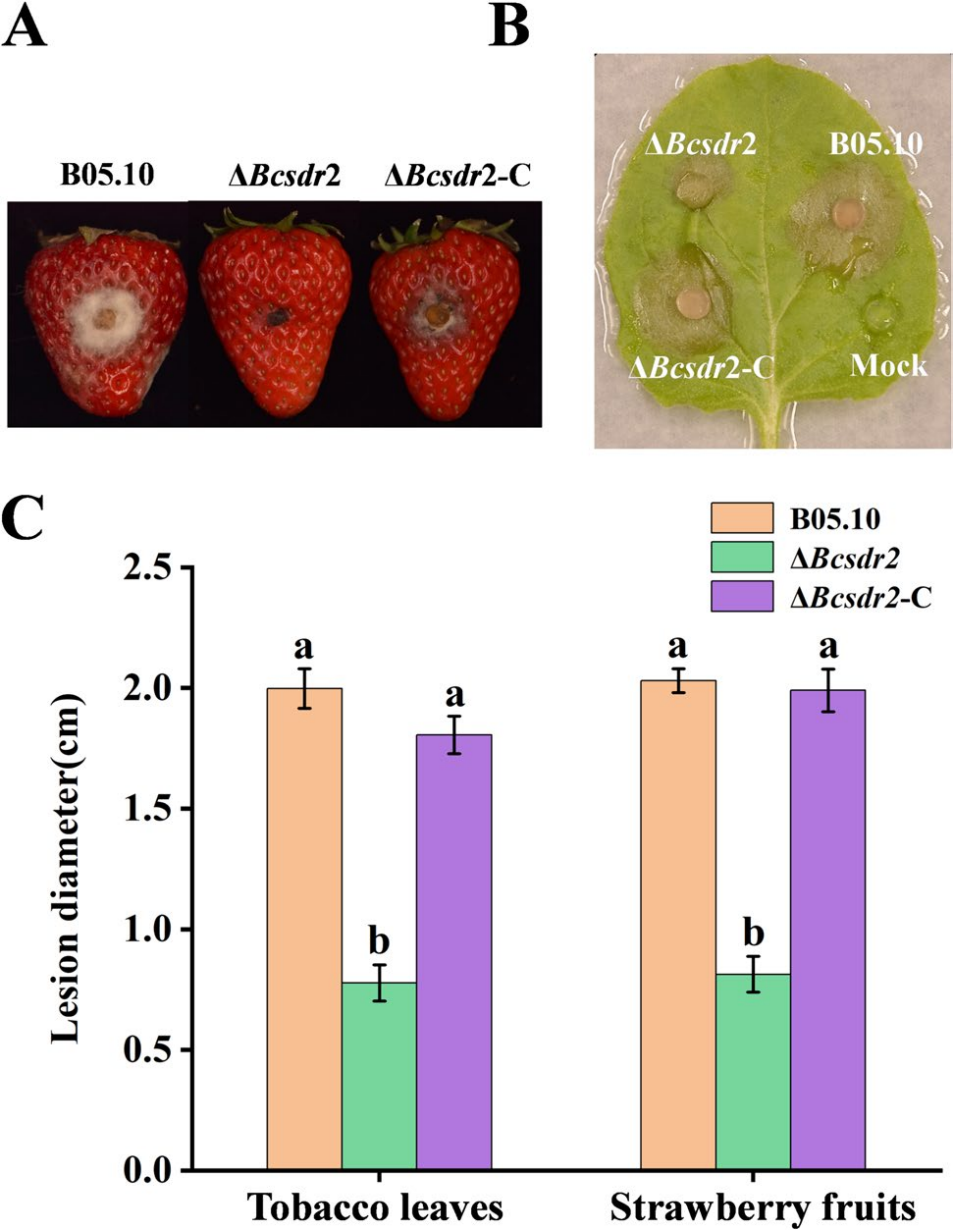
Effects of Bcsdr2 deletion on mycelial infection and pathogenicity. Bars represent standard errors from three replicates. Values on bars followed by different letters indicate significant differences at *p* = 0.05. **A** and **B** Disease symptoms caused by each strain on strawberry fruits wounded and tobacco leaves. Images were captured at 96 h after inoculation. **C** Pathogenicity on tobacco leaves and strawberry fruits after 96 h of incubation

To determine whether Bcsdr2 is involved in regulating pathogenicity in *B. cinerea*, strawberry fruits and tobacco leaves were inoculated with Δ*Bcsdr2* mutants. Compared with the WT strain, Δ*Bcsdr2* mutants exhibited reduced virulence in different hosts (Fig. 4 A and B). At 96 h, tobacco leaves inoculated with Δ*Bcsdr2* mutants displayed small lesions, while WT-inoculated leaves had an average lesion size of 0.78 cm and 2.01 cm for tobacco leaves and strawberry fruits, respectively. Similarly, lesion size was considerably decreased on Δ*Bcsdr2* mutant-inoculated strawberry fruits compared with WT-inoculated fruits (Fig. 4C). Complemented strain Δ*Bcsdr2*-C exhibited almost the same level of virulence as the WT strain. These results suggest that Bcsdr2 plays a crucial role in the virulence of *B. cinerea*.

### Effects of Bcsdr2 deletion on sensitivity to abiotic stresses and pathogenicity factors

**Fig. 5:**
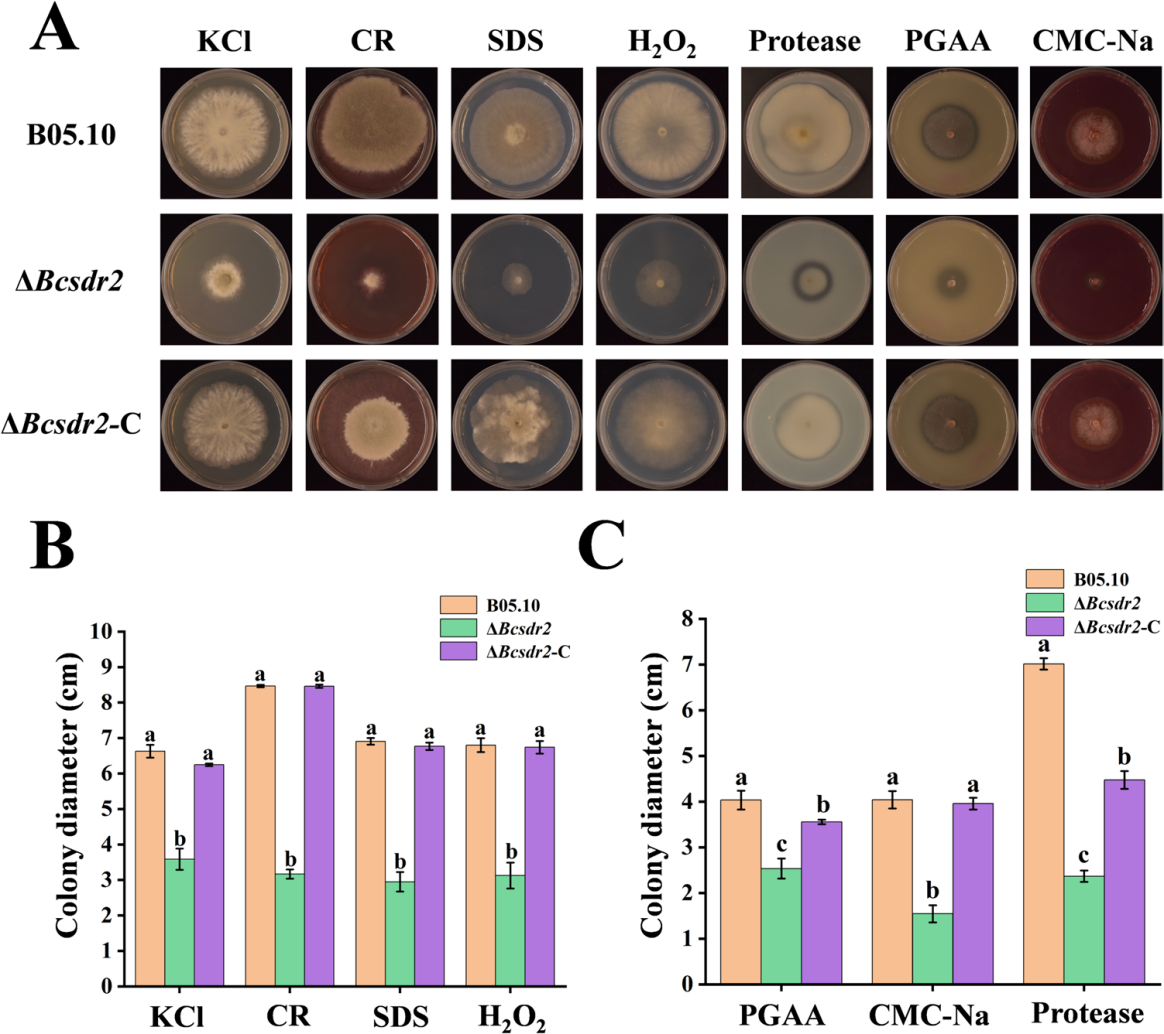
Sensitivity of Δ*Bcsdr2*, B05.10 and Δ*Bcsdr2*-C strains to abiotic stresses. Bars represent standard errors from three replicates. Values on bars followed by different letters indicate significant differences at *p* = 0.05. **A** All strains were grown on PDA plates amended with KCl, CR, SDS or H_2_O_2_ at the indicated concentrations at 20 °C for 2 days, and with skimmed milk powder, polygalacuronic acid or carboxymethyl cellulose at 20 °C for 3 days. **B** Sensitivity of Δ*Bcsdr2*, B05.10 and Δ*Bcsdr2*-C strains to KCl, CR, SDS and H_2_O_2_. **C** Ability of Δ*Bcsdr2*, B05.10 and Δ*Bcsdr2*-C strains to produce polygalacturonase, cellulases and proteases

The results of mycelial response assays showed that compared with Δ*Bcsdr2*-C and B05.10, Δ*Bcsdr2* mutants exhibited suppressed mycelial growth in the presence of KCl, CR, SDS and H_2_O_2_, and the ability to produce proteases, polygalacturonase and cellulases was significantly reduced (Fig. 5A). These results suggest that disruption of Bcsdr2 may have marginal effects on mycelial growth in response to abiotic stresses, and pathogenicity factor production capacity may also be affected.

### Bcsdr2 deletion affects transcription and pathogenicity-related genes

**Fig. 6:**
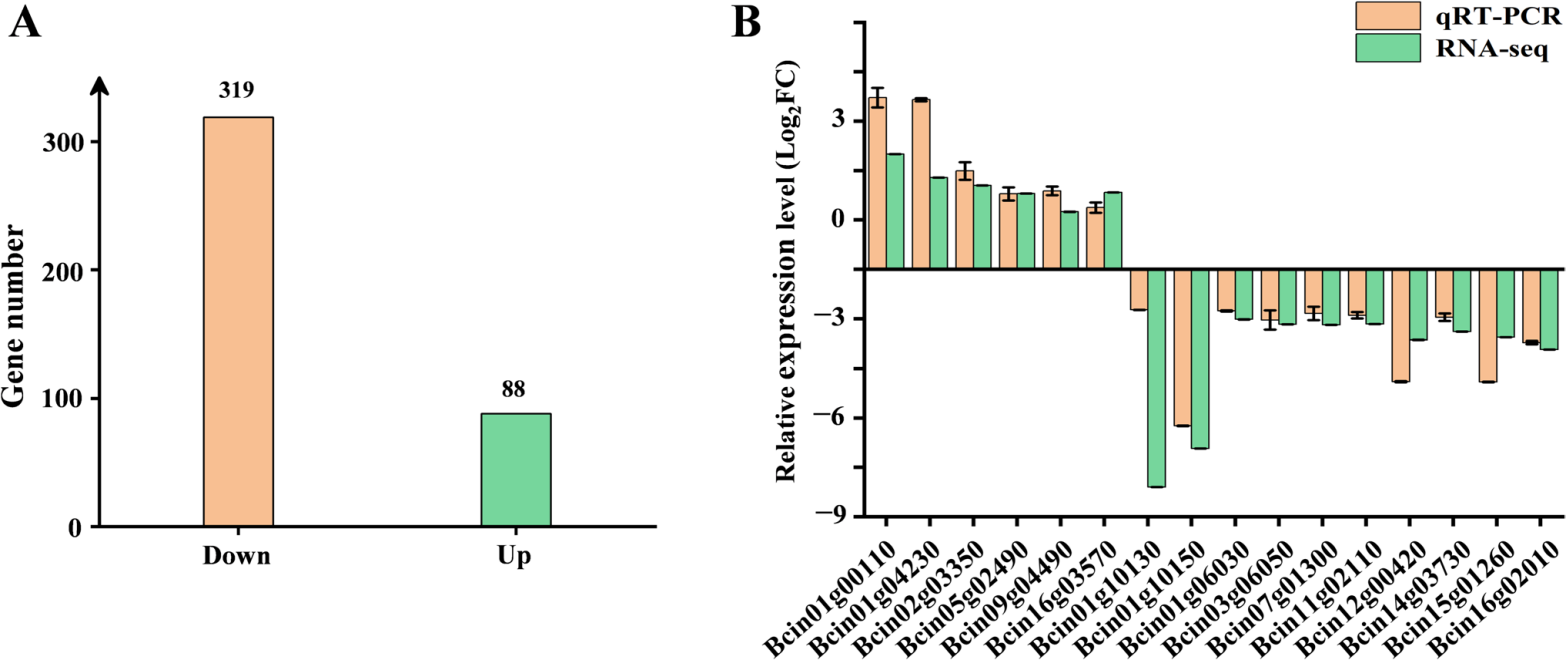
RNA-seq analysis of Δ*Bcsdr2* deletion strains. **A** Number of up- and downregulated genes (*p* < 0.05, fold change > 2) in Δ*Bcsdr2* strains compared with WT B05.10. **B** qRT-PCR of Δ*Bcsdr2* transcriptome DEGs

We performed an RNA-Seq analysis to identify genes that might exhibit changes in regulation affected by Bcsdr2 in *B. cinerea*. Three biological replicates with mRNA isolated from WT B05.10 and Δ*Bcsdr2* strains were performed, and 319 downregulated and 88 upregulated (fold change > 2, *p* < 0.05) genes were identified in Δ*Bcsdr2* compared with B05.10 (Fig. 6A).

Functional annotation of DEGs via GO analysis was performed to identify genes belonging to molecular function, cellular component and biological process categories (Supplemental Fig. S1). Among them, oxidoreductase activity/acting on paired donors, with incorporation or reduction of molecular oxygen/iron ion binding, organelle envelope/mitochondrial outer membrane/organelle outer membrane and oxidation–reduction process/ribosome biogenesis/ribonucleoprotein complex biogenesis, was the main molecular function, cellular composition and biological process subcategories, respectively. These results showed that growth and pathogenicity defects caused by the absence of Bcsdr2 in the B05.10 strain were closely related to these functions.

In addition, enriched DEGs can be functionally classified into metabolism, cellular processes and genetic information processing. Among the metabolic pathways belonging to these three categories, the top ten metabolic pathways of enriched DEGs were metabolic pathways, ribosome biogenesis in eukaryotes, biosynthesis of secondary metabolites, biosynthesis of antibiotics, purine metabolism, RNA polymerase, propanoate metabolism, 2-oxocarboxylic acid metabolism, valine, leucine and isoleucine degradation and amino sugar and nucleotide sugar metabolism, respectively (Supplemental Fig. S2). These metabolic pathways may be closely related to the growth and pathogenicity defects of Δ*Bcsdr2*.

To verify the reliability of DEGs identified from transcriptome sequencing, qRT-PCR was performed on the remaining Δ*Bcsdr2* RNA samples used for transcriptome analysis. qRT-PCR validation was performed by randomly selecting 16 growth- and pathogenicity-associated genes. Their melt curves are attached to the supplement figures (Fig.S3-Fig. 19). These genes encode proteins that participate in growth regulation (Bcin01g10130, Bcin01g06930, Bcin11g02110), the synthesis of substances and catalytic reactions of enzymes (Bcin02g03350, Bcin01g10150, Bcin12g00420) and virulence and stress factors (Bcin01g04230, Bcin01g00110, Bcin07g01300, Bcin14g03730). The qRT-PCR results were consistent with the RNA-seq results (Fig. 6B), confirming the reliability of the RNA-seq data.

## Discussion

Short-chain dehydrogenases (SDRs) are NAD(P)-dependent oxidoreductases that participate in the metabolism of various specific substrates in organisms through oxidation–reduction, isomerisation and cleavage, thereby regulating biochemical reactions and physiological processes (Roth et al. 2018; Cui et al. 2019). SDRs are closely associated with plant growth and development and stress response mechanisms (Stavrinides et al. 2018), as well as pathogenic processes of pathogens. Studies have found that the SDR MoSDR1 inhibits spore formation and germination of rice blast fungus, suppresses the development of invasive structures and reduces the pathogenicity of the rice blast fungus (Kwon et al. 2010). SDRs can also induce systemic resistance in plants and hinder the invasion of plant fruits by pathogens (Hwang et al. 2012). Recent discoveries have revealed that SDRs are capable of sensing the redox state in metabolism and participating in transcription or RNA processing, further extending the functional scope of this superfamily of proteins (Zhao 2018).

To explore the function of membrane protein Bcsdr2, a member of the SDR family, we first disrupted the *Bcsdr2* gene and characterised the resulting mutant, which showed severe defects in hyphae grow and pathogenicity. We therefore speculated that Bcsdr2 might be involved in regulating hyphae growth and pathogenicity-related genes in its fungal host. Bcsdr2 is a vital virulence determinant since deletion of the *Bcsdr2* gene also compromised the penetration ability of *B. cinerea*, indicating that the reduced virulence of the Δ*Bcsdr2* mutant was likely due, at least in part, to defective penetration of host cells. In addition, the ability of the Δ*Bcsdr2* mutant strain to produce cellulases and proteases was significantly reduced, which is another important factor of pathogenicity (He 2014; Hou 2019). Based on the above results, the Bcsdr2 protein appears to be an important virulence factor of *B. cinerea*. However, the regulatory mechanisms involving Bcsdr2 remain poorly understood, and further research such as a comparative analysis of transcription profiles could provide valuable information.

We used transcriptome sequencing technology to compare and analyse transcriptional regulation differences and DEGs of *∆Bcsdr2* mutant strains following culture for 72 h, and 407 DEGs were screened. GO functional analysis showed that in molecular function and biological process categories, DEGs resulting from loss of the *Bcsdr2* gene were mostly involved in oxidoreductase activity and oxidation–reduction process, respectively. Among the cellular component terms, the most enriched were linked to cellular structure and membrane, consistent with the fact that deletion of the *Bcsdr2* gene affected biofilm integrity based on TEM observations. Our RNA-seq analysis results suggest that global changes in genes involved in metabolic pathways, biosynthesis of secondary metabolites, ribosome biogenesis in eukaryotes and protein processing in endoplasmic reticulum are likely to underlie this defect.

The reliability of transcriptome data was verified by qRT-PCR analysis of nine growth- and pathogenicity-related genes. Mitochondria and essential organelles in organisms and the primary site for energy production and aerobic respiration. Phosphatidylethanolamine (PE), synthesised from phosphatidylserine by the enzyme encoded by the *psd* gene, is a non-bilayer phospholipid that helps maintain the shape and function of mitochondria and is involved in regulating the growth, development and virulence of *Fusarium graminearum* (Tang et al. 2021; Gok et al. 2022). Cox17 is an essential protein for cytochrome c oxidase within the mitochondria that plays a regulatory role in the structure of mitochondrial membranes (Vanišová et al. 2019; Ding et al. 2022). In the present study, the relative expression level of *Bcpsd* was downregulated 0.82-fold while the relative expression level of *Bccox17* was upregulated 1.99-fold. TEM revealed that loss of the membrane protein Bcsdr2 led to mitochondrial swelling, disappearance of the outer membrane and dissolution of the cristae. The significant changes in the expression of the aforementioned genes may be important factors affecting mitochondrial morphology and function. The ribosome, an essential molecular machine responsible for protein synthesis, is composed of the 40S small subunit and the 60S large subunit in eukaryotes. Research has found that Nmd3 acts as a structural mimic of eIF5A and activates the cpGTPase Lsg1 during biogenesis of the ribosome 60S large subunit, thereby influencing its synthesis in mitochondria (Malyutin et al. 2017). By contrast, the RNA-binding protein Nob1 is necessary for the synthesis of the ribosome 40S small subunit (Fatica et al. 2004; Lamanna and Karbstein 2009). In the present work, the relative expression levels of *Bcnmd3* and *Bcnob1* were downregulated 2.27-fold, indicating that the loss of membrane protein Bcsdr2 affects the biogenesis of ribosomal subunits. Normal growth and development of microorganisms are essential for their life activities. Studies have found that deletion or downregulation of *BOA11*, *HMT1* and *DUG2* genes can inhibit the growth of strains and reduce conidiation (Porquier et al. 2019; Li et al. 2020; Reza and Sanyal 2022). In this study, expression levels of *BcBOA11*, *Bchmt1* and *Bcdug2* were downregulated to varying degrees, indicating a close relationship between the decreased growth rate and morphological changes in Δ*Bcsdr2* and the aforementioned genes. The ability to respond to stressful environments is an important indicator of microbial vitality. Studies have found that *met16*, *fap7* and *carA* genes are important factors in microbial response to oxidative stress (Juhnke et al. 2000; Lage et al. 2019; Buvelot et al. 2021; Luo and Xu 2021). In the present study, the relative expression level of *Bcmet16* was upregulated 3.43-fold, while expression levels of *Bcfap7* and *BccarA* were downregulated 1.03-fold and 0.84-fold, respectively, consistent with the reduced ability of Δ*Bcsdr2* to respond to stress conditions. There were also some studies that suggested that knockout genes can influence the sensitivity to abiotic stresses of *Botrytis* (Schamber, et al. 2010; Tundo et al. 2020). Therefore, the observed changes in pathogenicity, growth and development, stress responses and mitochondrial morphology for the Δ*Bcsdr2* strain are closely associated with the differential expression of the aforementioned genes.

Deletion of Bcsdr2 significantly reduced the ability of hyphae to produce infection structures, and the ability to produce acids, cellulases and proteases was also significantly diminished, which further indicates that Bcsdr2 may reduce the pathogenicity of strains, and transcriptome and quantitative PCR results further supported these conclusions. Therefore, we preliminarily concluded that the absence of Bcsdr2 can reduce the growth rate and pathogenicity of *B. cinerea*, while increasing sensitivity to environmental stress.

This further indicates that wuyiencin disrupts the integrity of *B. cinerea* biofilms by regulating the expression of membrane protein Bcsdr2, thereby inhibiting hyphal growth and development, and decreasing the invasive capacity and pathogenicity of the strain.

In summary, membrane protein Bcsdr2 is involved in regulating *B. cinerea* biofilm integrity, hyphal growth and virulence.

## Supplementary Information

Below is the link to the electronic supplementary material.

Supplementary file1 (PDF 3593 KB)

## Data Availability

Data will be made available on request, and the transcriptome sequencing data was deposited in NCBI with a number of PRJNA1071901.
