# Supplementary material for: Membrane protein Bcsdr2 mediates biofilm integrity, hyphal growth and virulence of Botrytis cinerea
Source: Appl Microbiol Biotechnol. 2024 Jun 28;108:398. doi: 10.1007/s00253-024-13238-8 (PMC11213811; doi:10.1007/s00253-024-13238-8)
Supplement: Supplementary file 1 [file 253_2024_13238_MOESM1_ESM.pdf]

Journal name:

Applied Microbiology and Biotechnology

Manuscript Title:

Membrane Protein Bcsdr2 Mediates Biofilm Integrity, Hyphal Growth and Virulence of *Botrytis cinerea*

Author name:

Wei Zhang[1,2], Yi Cao[3], Hua Li[4], Abdel-Hamied M. Rasmey[5], Kecheng Zhang[1], Liming Shi[1] and Beibei Ge[1]

The affiliation(s) and address(es) of the author(s)

[1] State Key Laboratory of Biology of Plant Diseases and Insect Pests, Institute of Plant Protection, Chi-nese Academy of Agricultural Sciences, 2 Yuanmingyuan West Road, 100193, Beijing, China

[2] Qian Xinan Branch of Guizhou Provincial Tobacco Company, 60 Ruijin Southern Road, 562499, Xingyi, China

[3] Guizhou Academy of Tobacco Science, 29 Longtanba Road, 550081, Guiyang, China

[4] School of Light Industry, Beijing Technology and Business University, 11 & 33 Fucheng Road, 100048, Beijing, China

[5] Botany and Microbiology Department, Faculty of Science, Suez University, Elsalam 1, Cairo-Suez road, 43721, Suez, Egypt

The e-mail address, telephone and fax numbers of the corresponding author

e-mail address: B.B.G., gebeibei@caas.cn; L.M.S., shiliming@caas.cn

telephone and fax numbers: 010-62815942

Supplement Figures

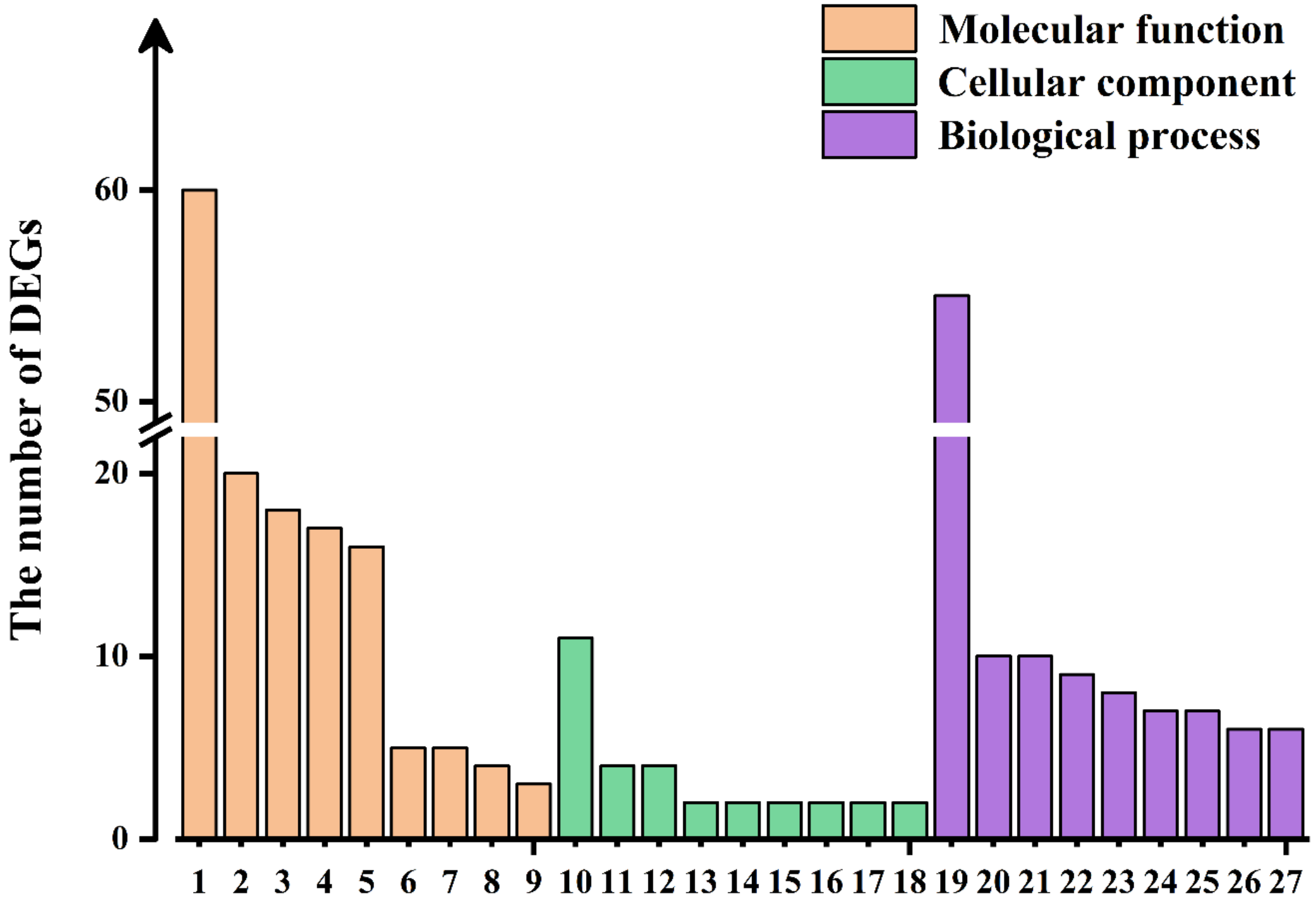

Fig. S1 GO functional classification of Δ*Bcsdr2* transcriptome DEGs.

Note: 1, oxidoreductase activity; 2, acting on paired donors, with incorporation or reduction of molecular oxygen; 3, iron ion binding; 4, tetrapyrrole binding; 5, heme binding; 6, inorganic anion transmembrane transporter activity; 7, anion transmembrane transporter activity; 8, molybdate ion transmembrane transporter activity; 9, oxidoreductase activity, acting on single donors with incorporation of molecular oxygen, incorporation of two atoms of oxygen; 10, organelle envelope; 11, mitochondrial outer membrane; 12, organelle outer membrane; 13, mRNA cap binding complex; 14, RNA cap binding complex; 15, mitochondrial proton-transporting ATP synthase complex, catalytic sector F(1); 16, proton-transporting ATP synthase complex, catalytic core F(1); 17, extracellular matrix; 18, elongator holoenzyme complex; 19, oxidation-reduction process; 20, ribosome biogenesis; 21, ribonucleoprotein complex biogenesis; 22, secondary metabolic process; 23, toxin metabolic process; 24, toxin biosynthetic process; 25, secondary metabolite biosynthetic process; 26, mycotoxin metabolic process; 27, rRNA processing.

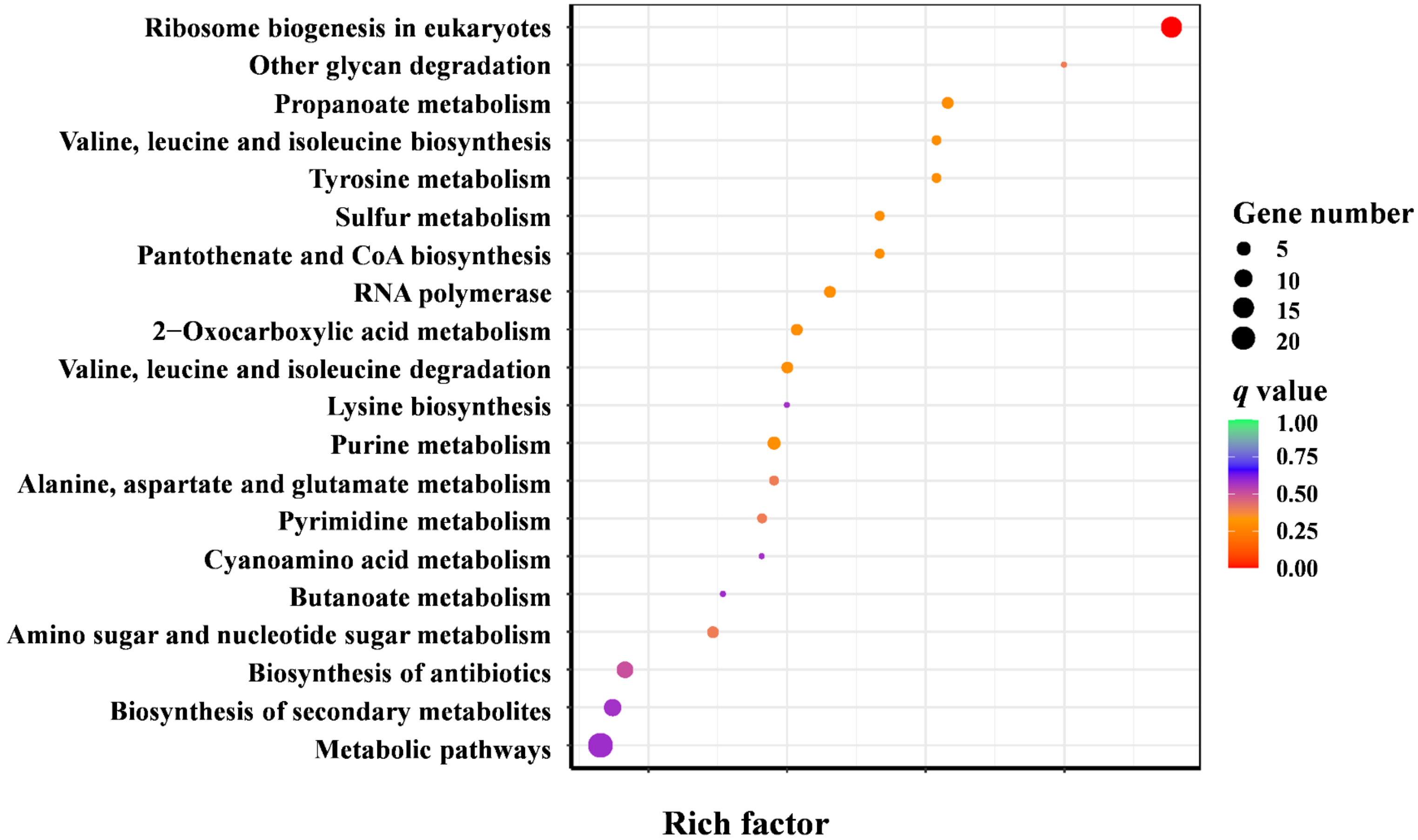

Fig. S2 KEGG enrichment analysis bubble diagram of the Δ*Bcsdr2* transcriptome.

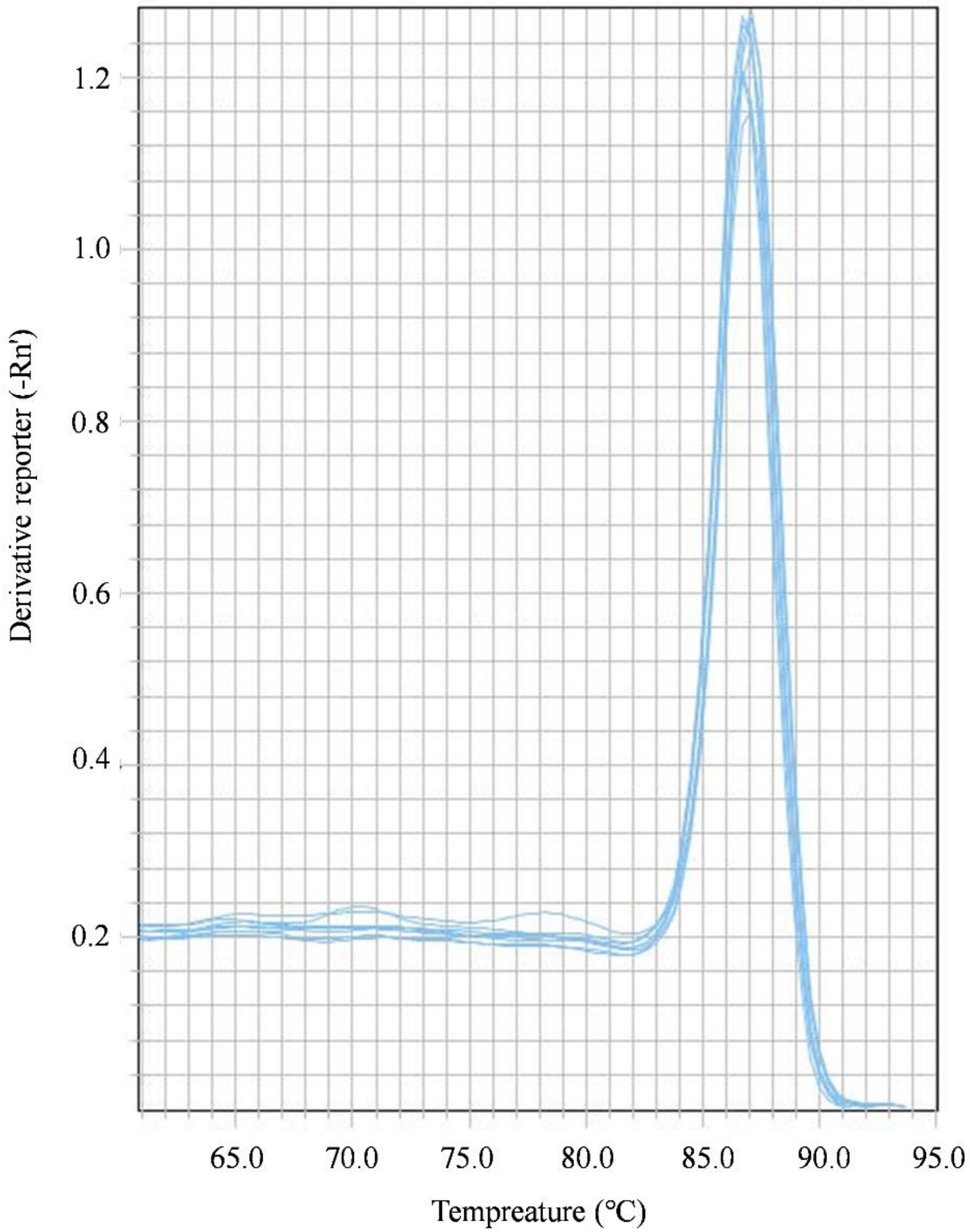

Fig. S3 Glycoside 65 melt curve

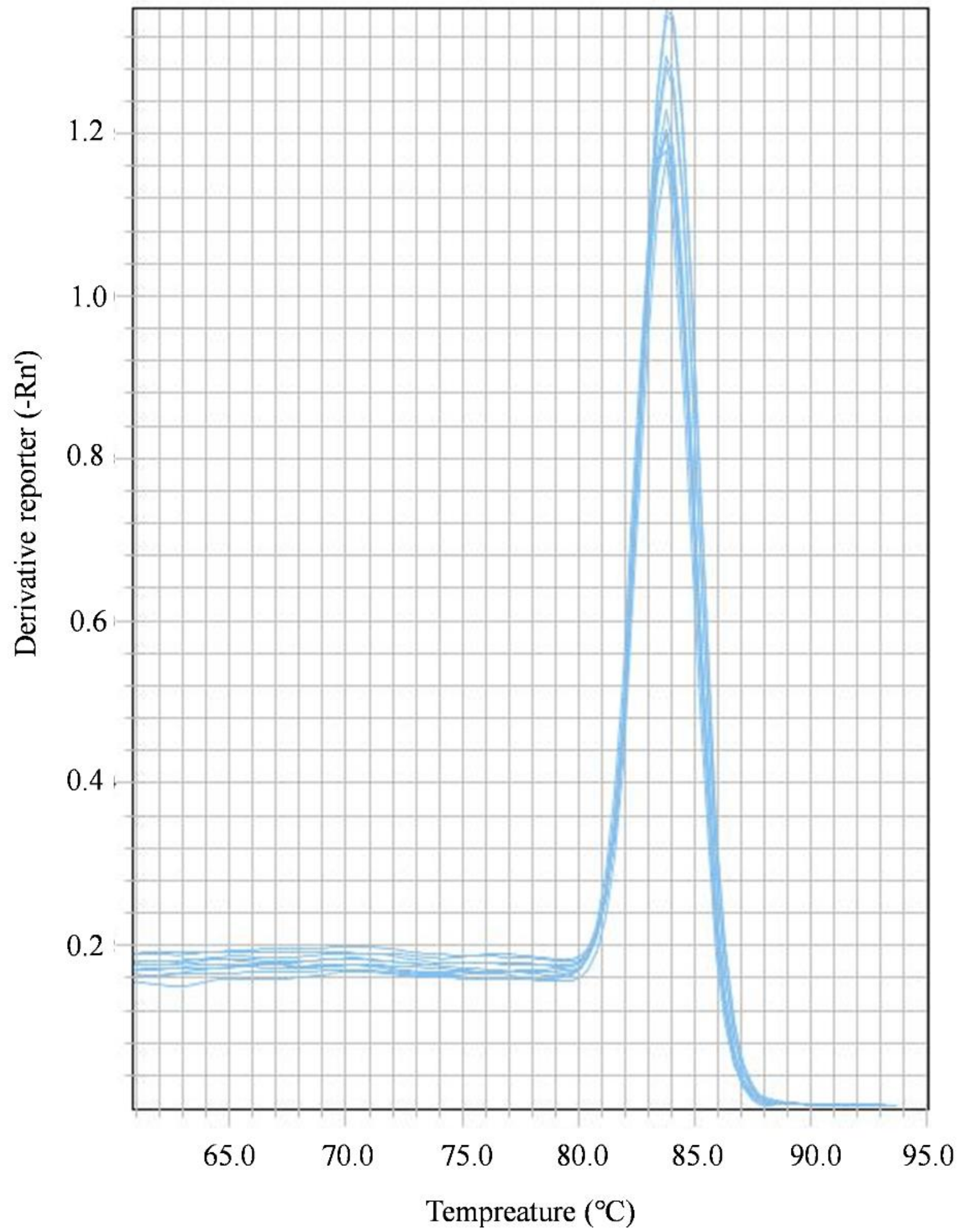

Fig. S4 Bcmet16 melt curve

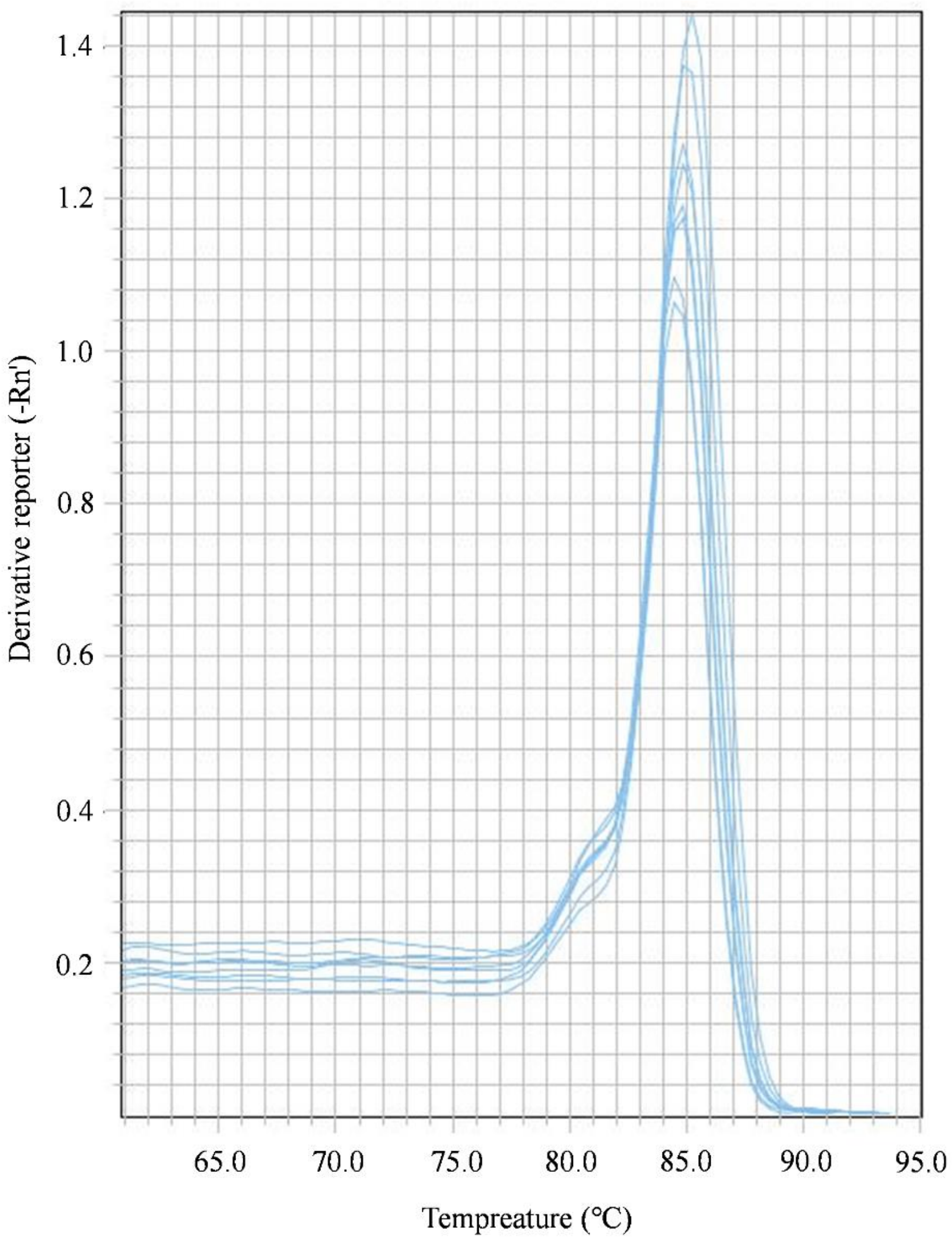

Fig. S5 BcCHSVII melt curve

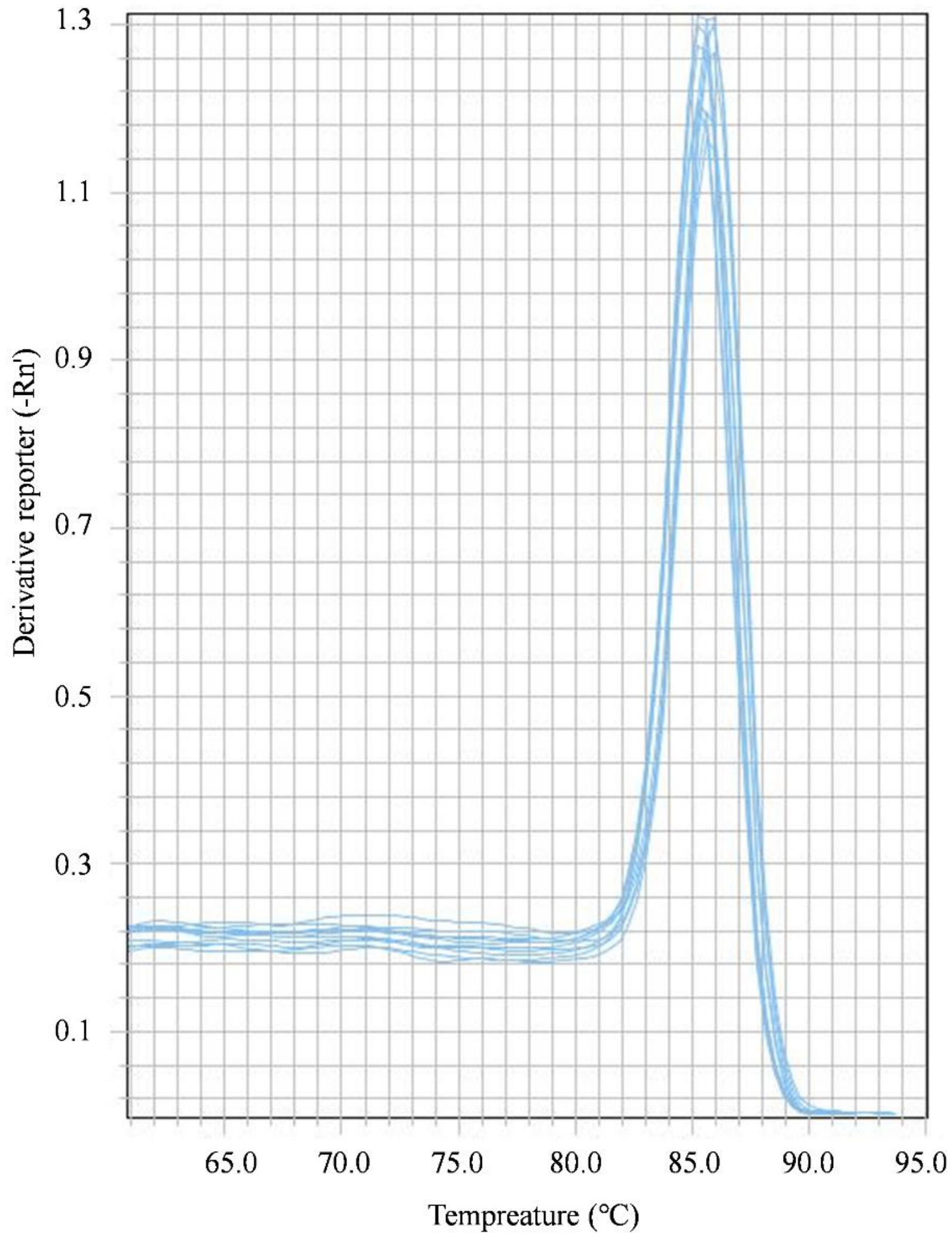

Fig. S6 Bcmxr1 melt curve

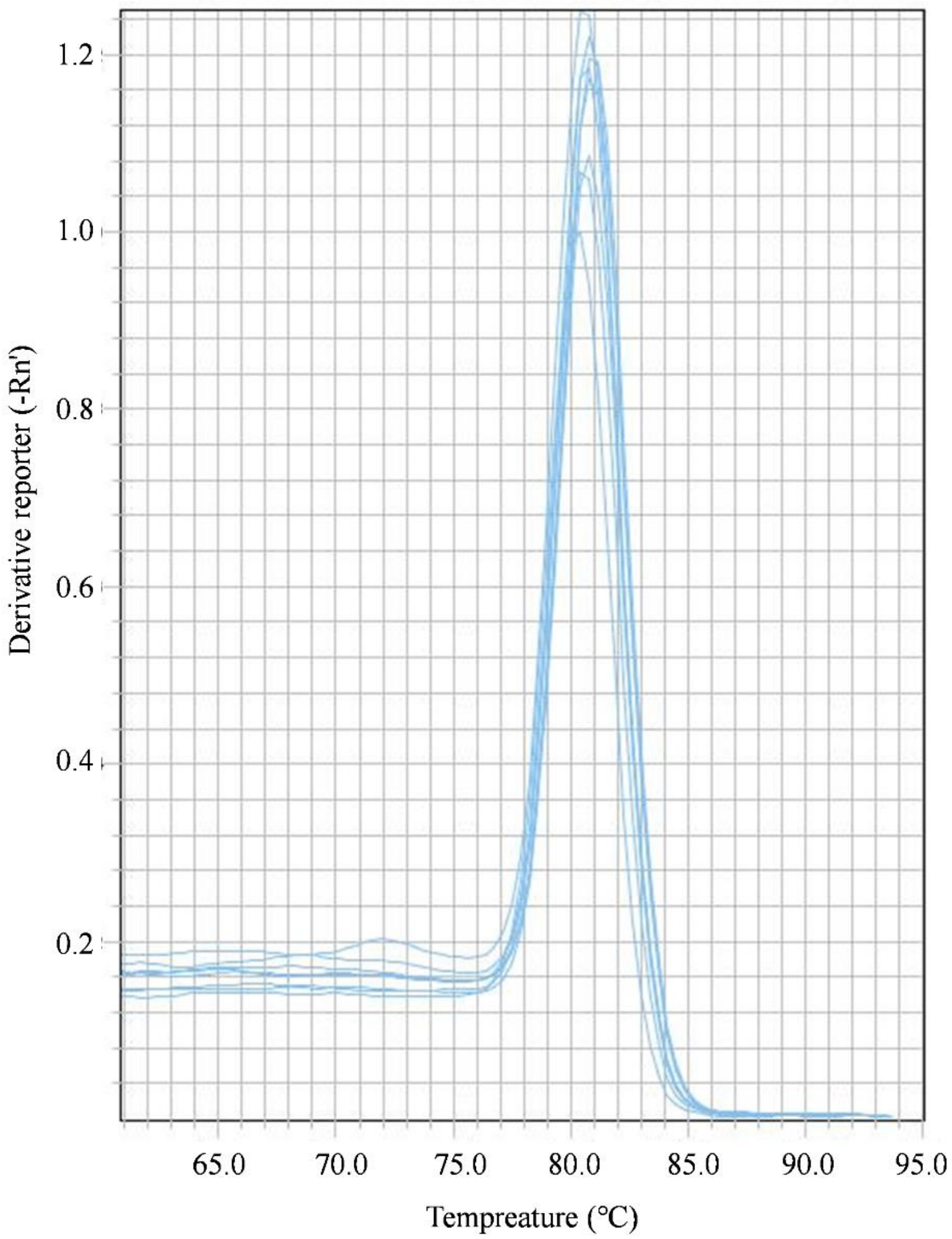

Fig. S7 Bcfap7 melt curve

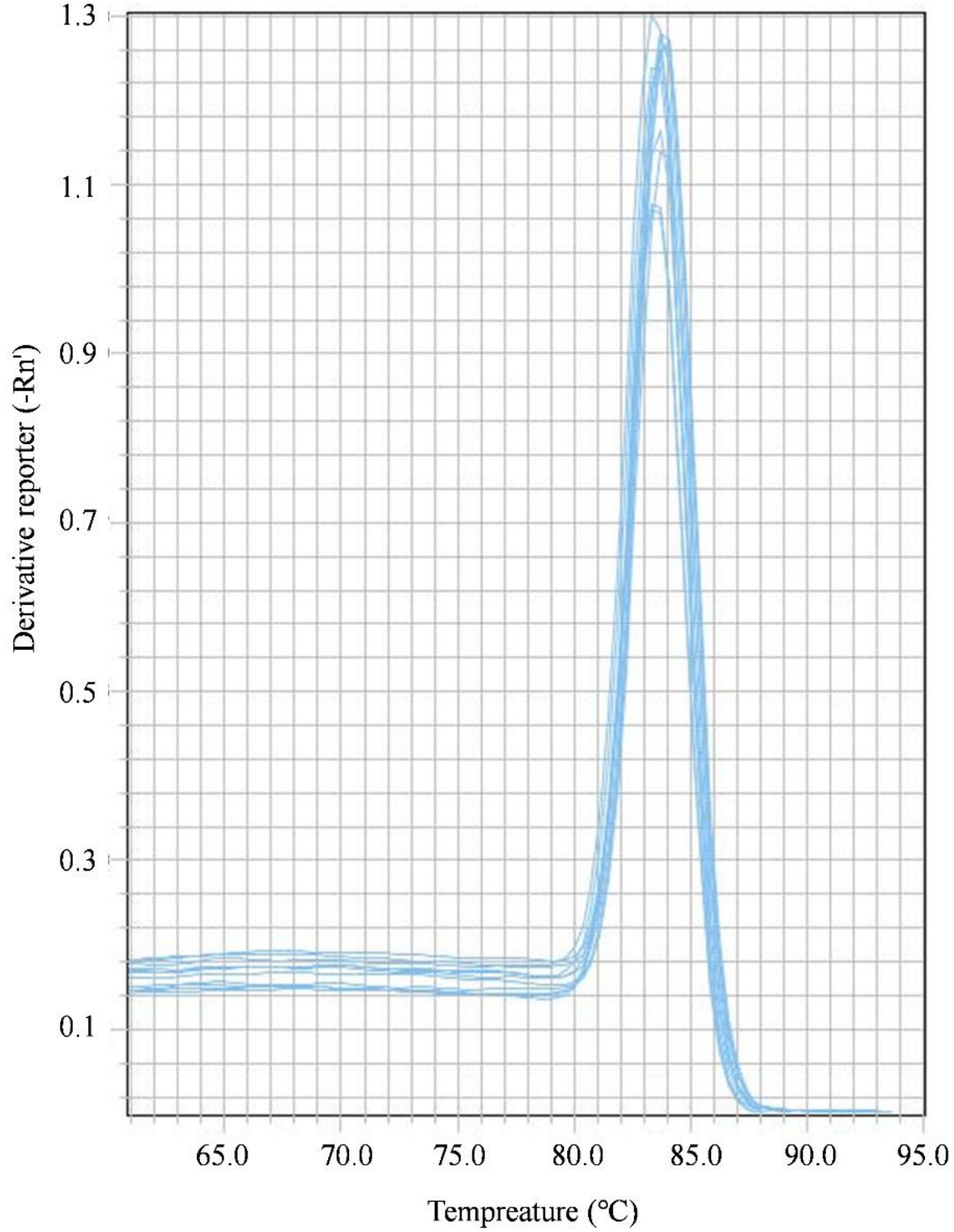

Fig. S8 Bchmt1 melt curve

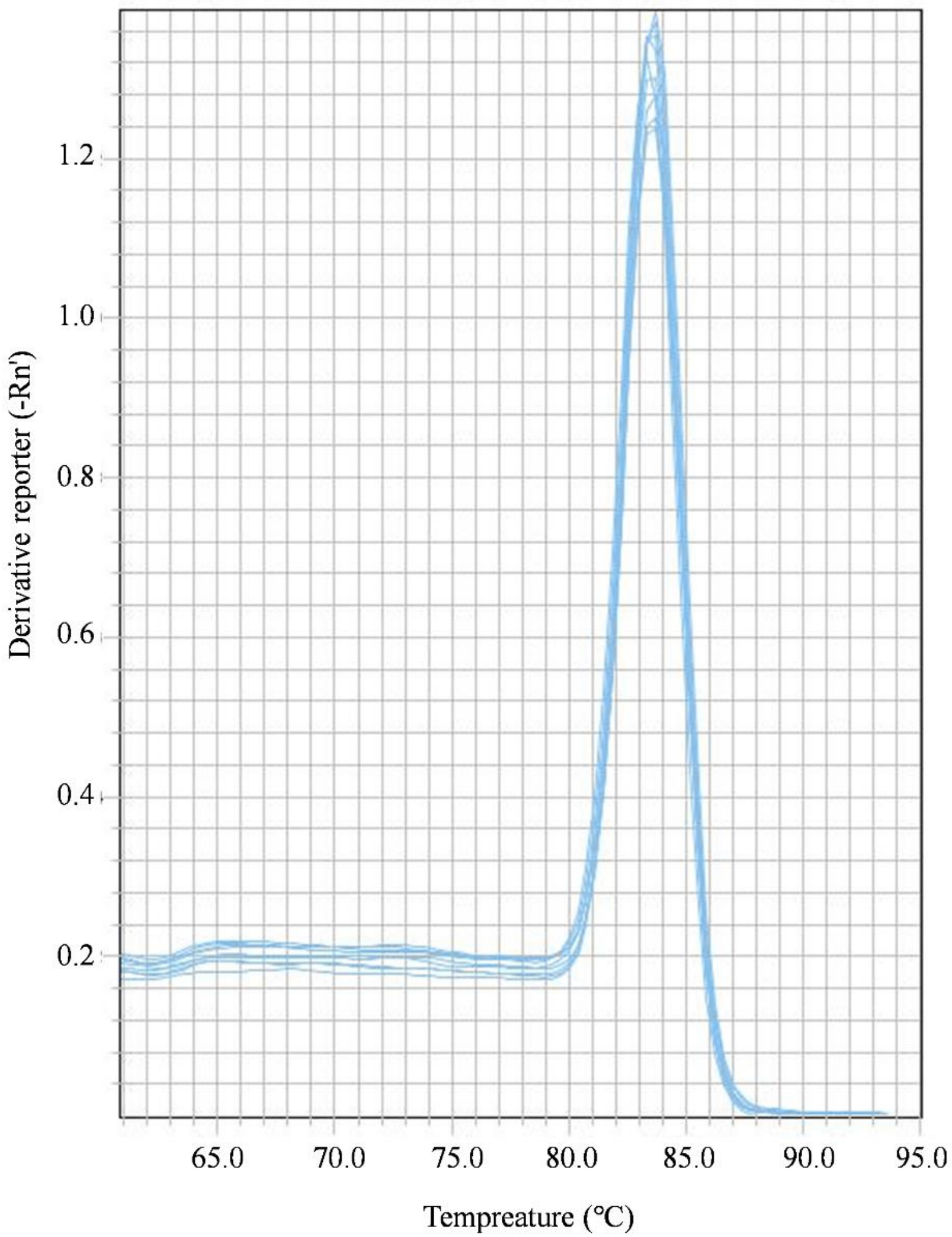

Fig. S9 BccarA melt curve

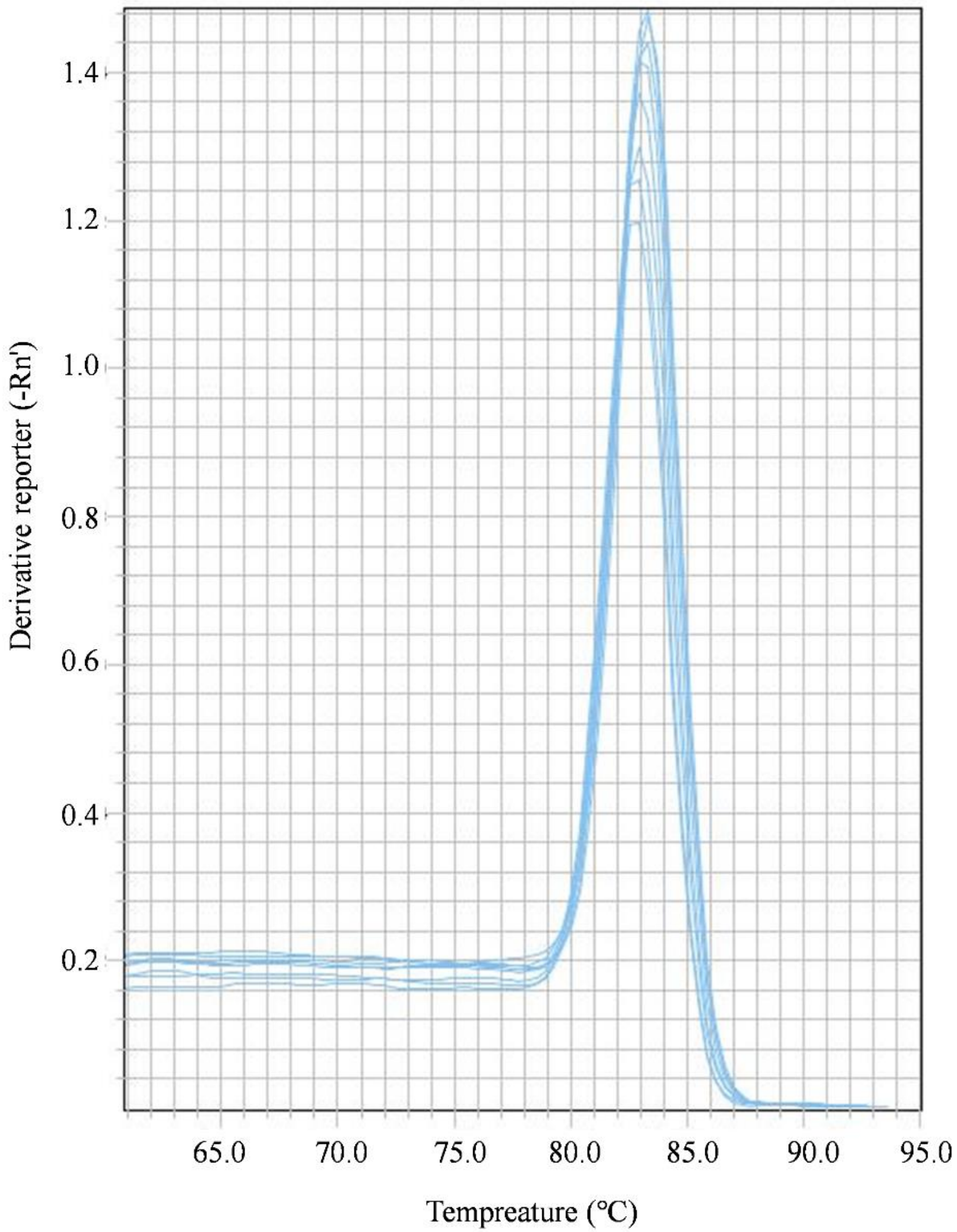

Fig. S10 Zinc C2H2 melt curve

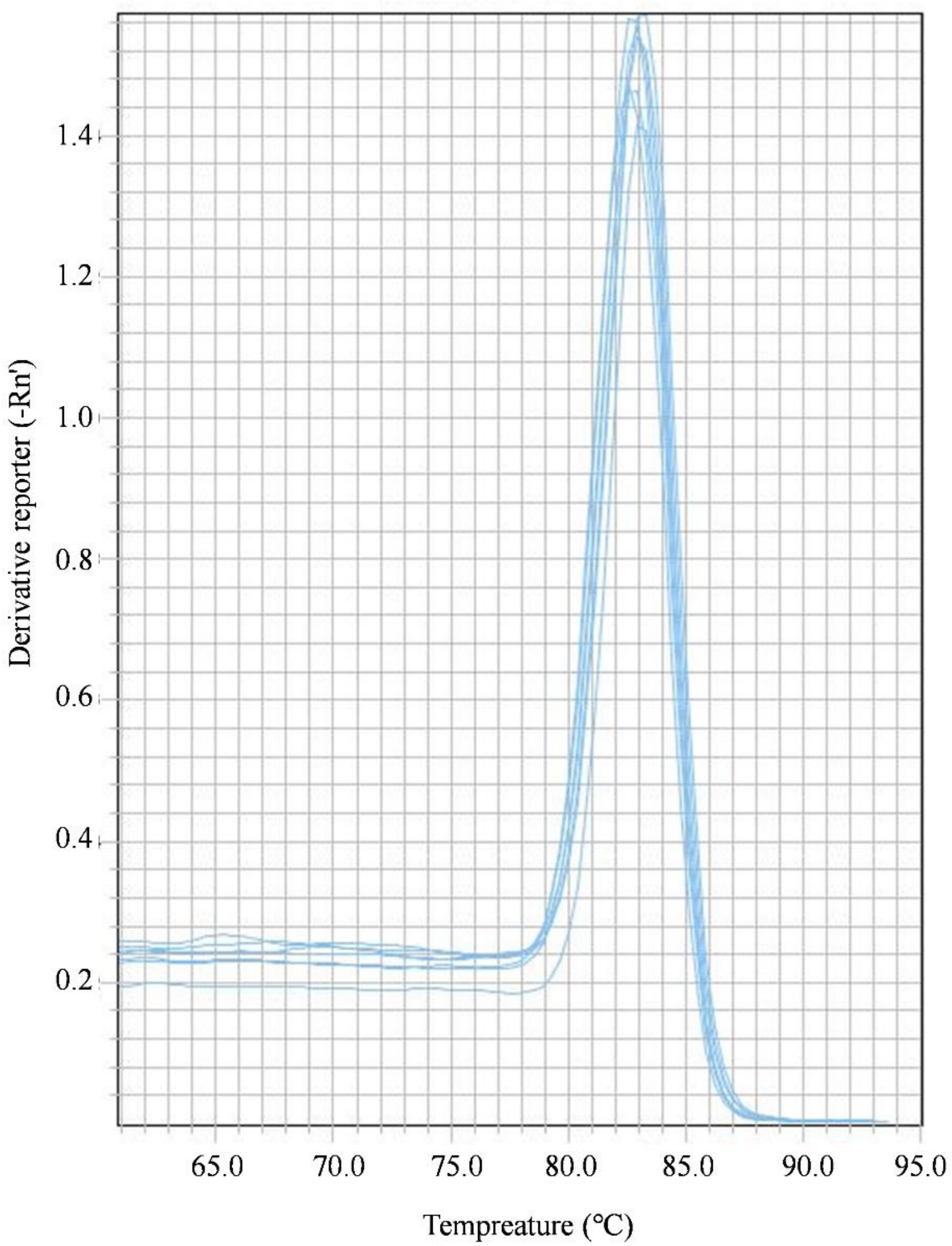

Fig. S11 Bcfes1 melt curve

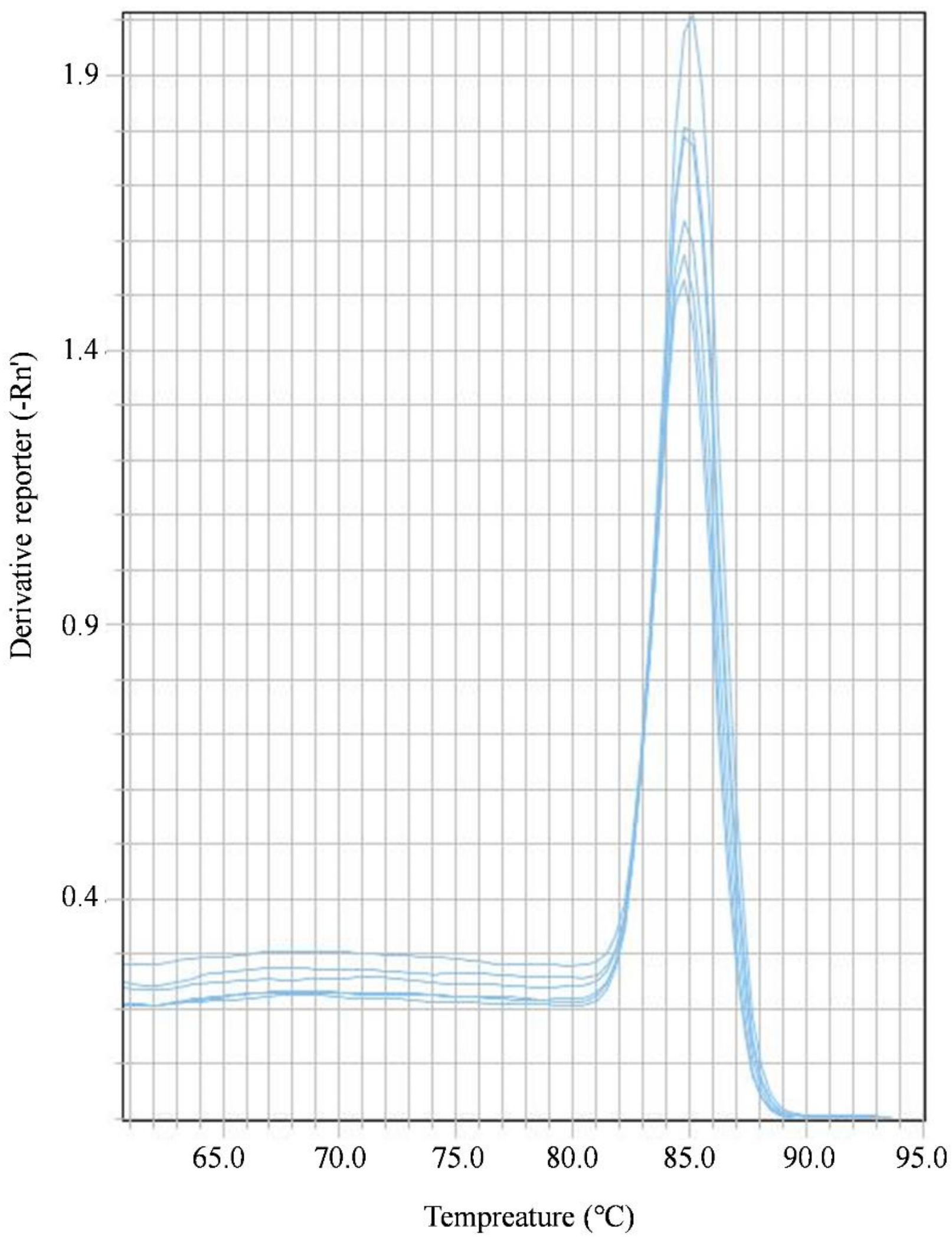

Fig. S12 Bcdug2 melt curve

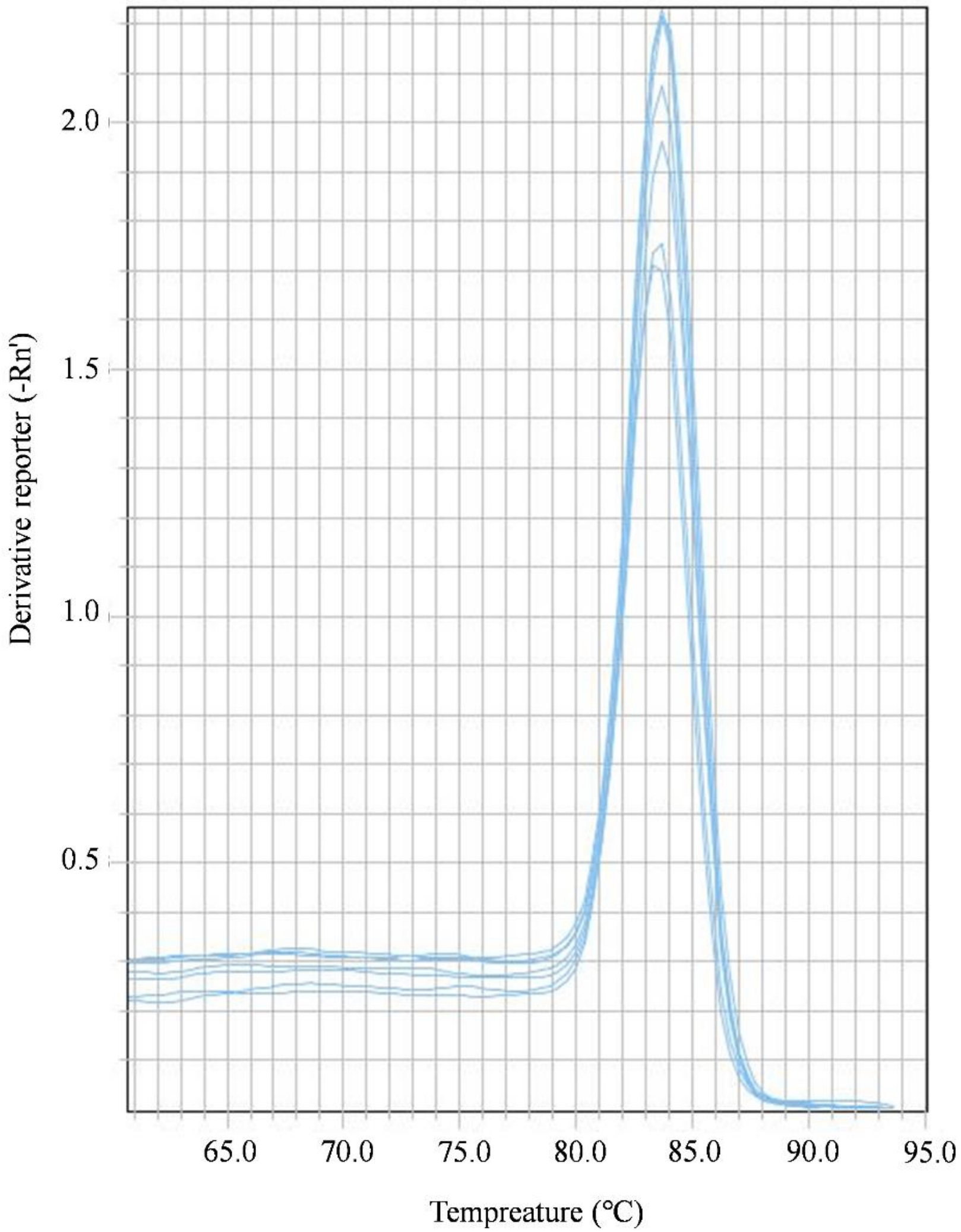

Fig. S13 Bccox17 melt curve

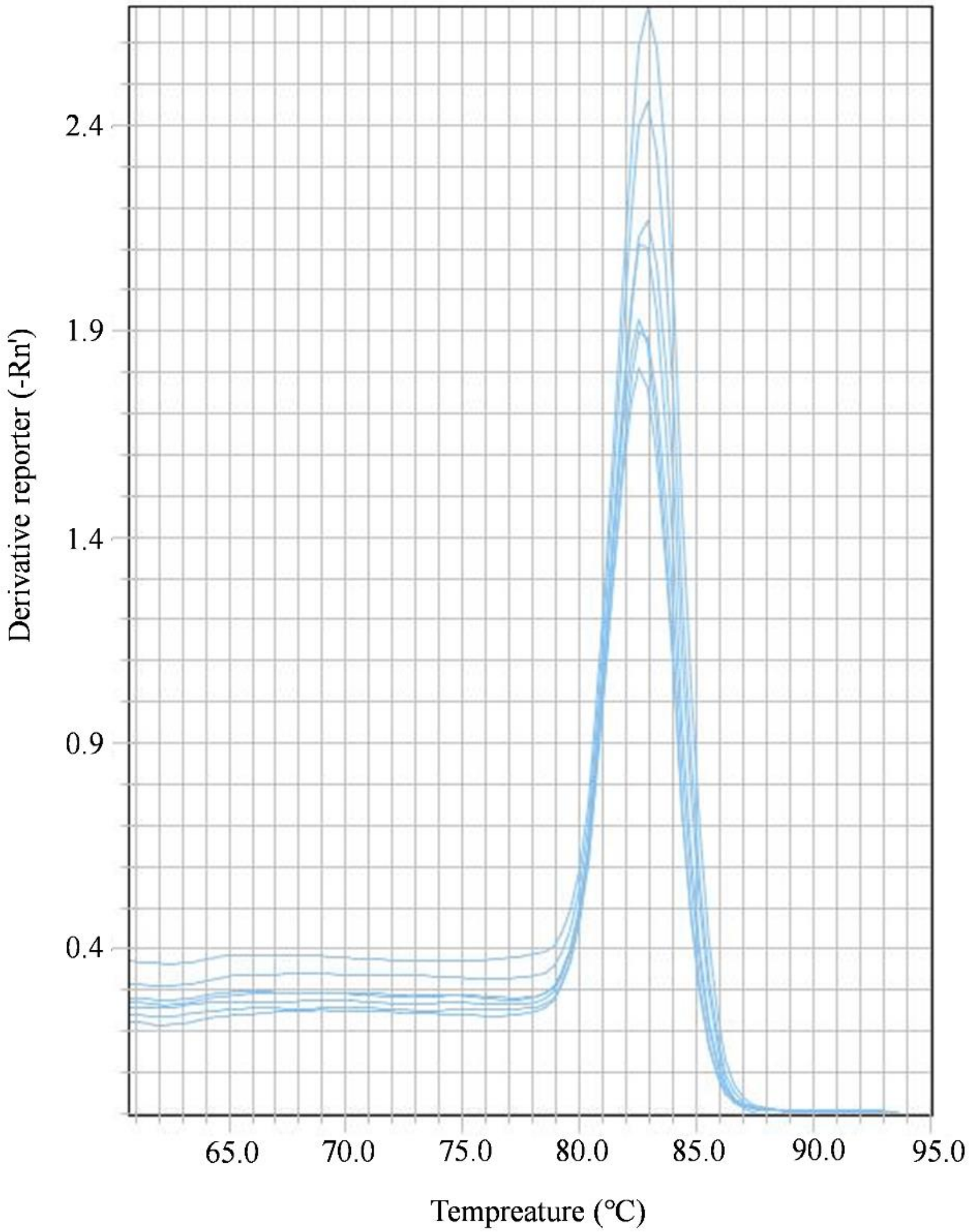

Fig. S14 Bcpsd melt curve

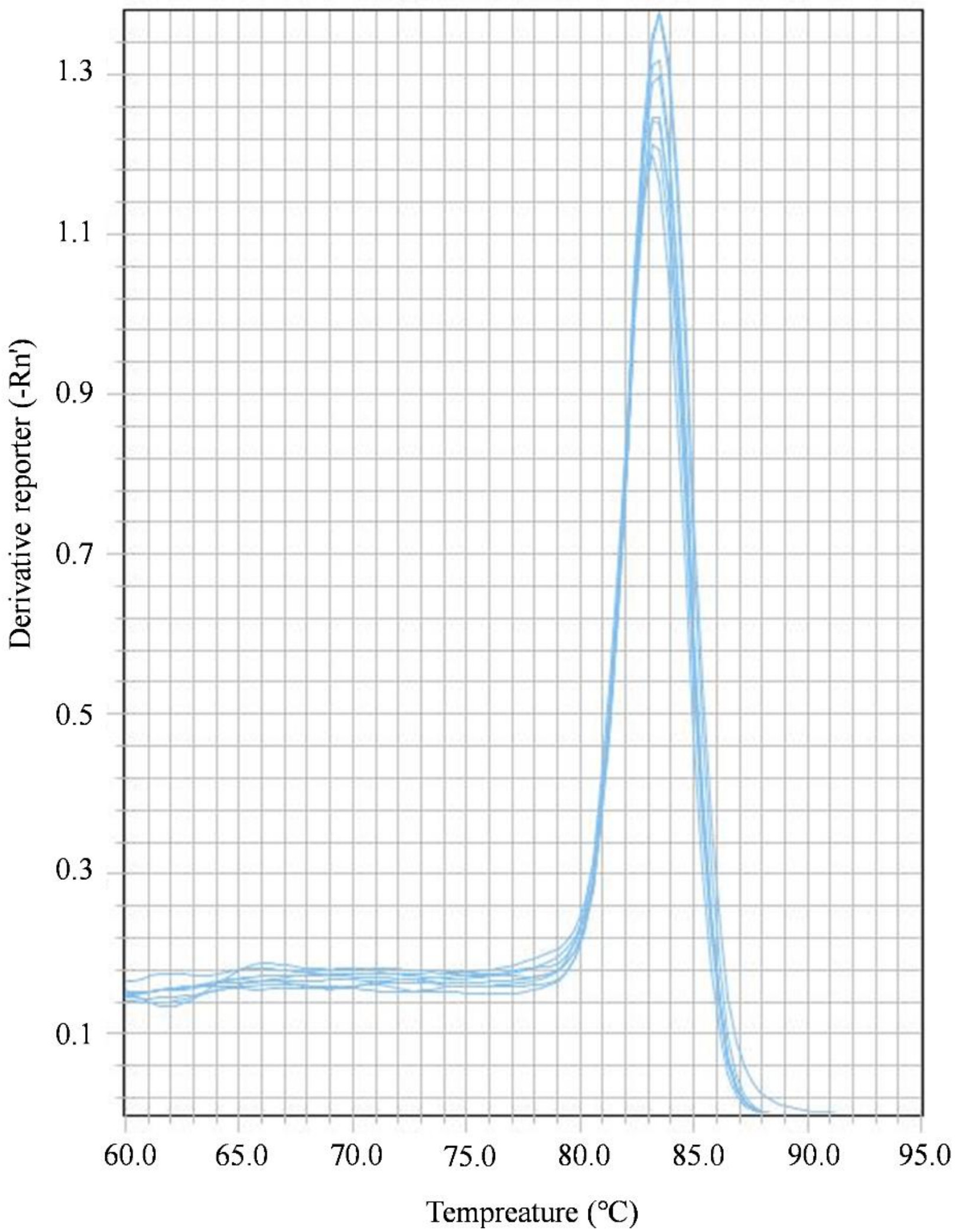

Fig. S15 BcNPRS melt curve

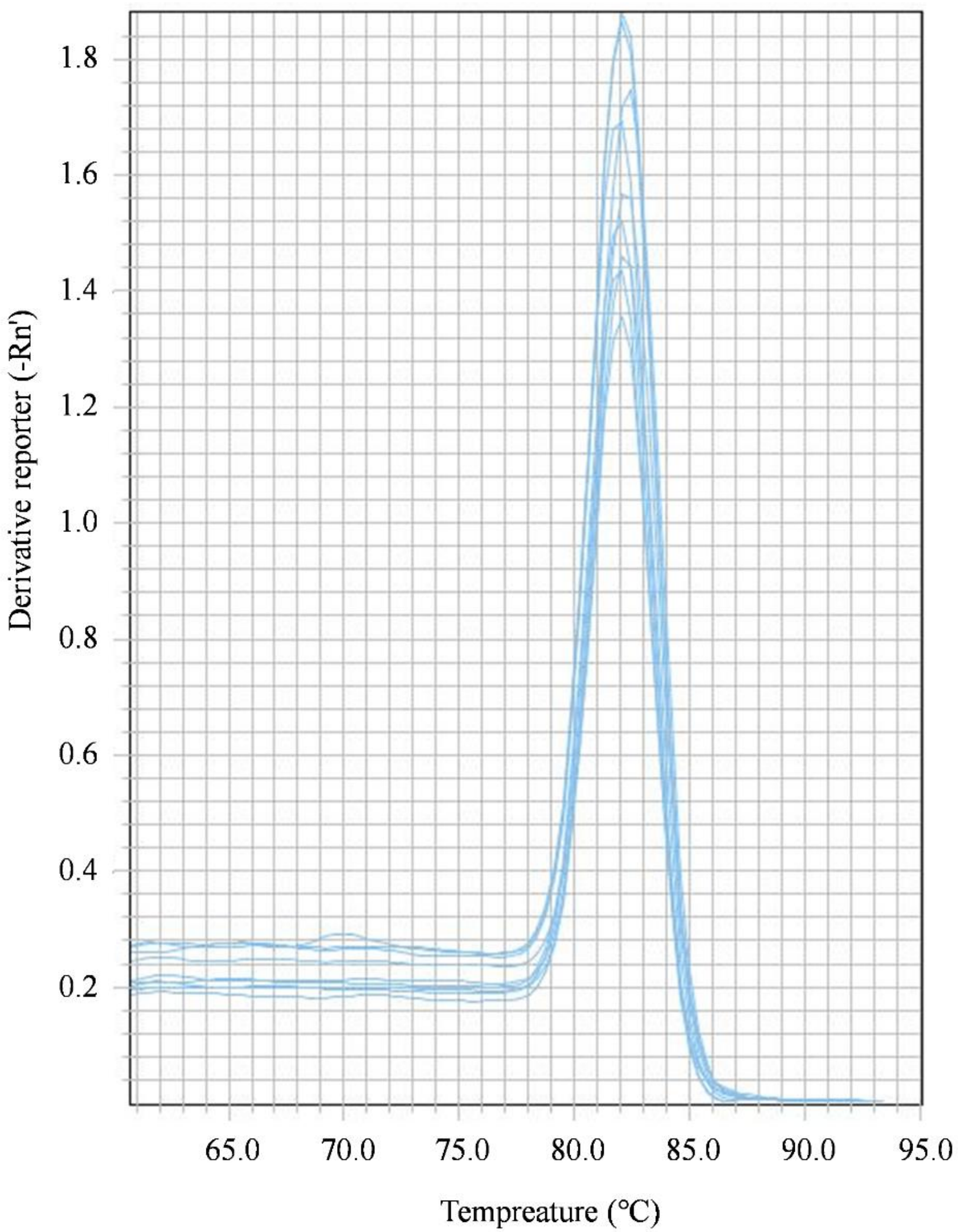

Fig. S16 Bcnmd3 melt curve

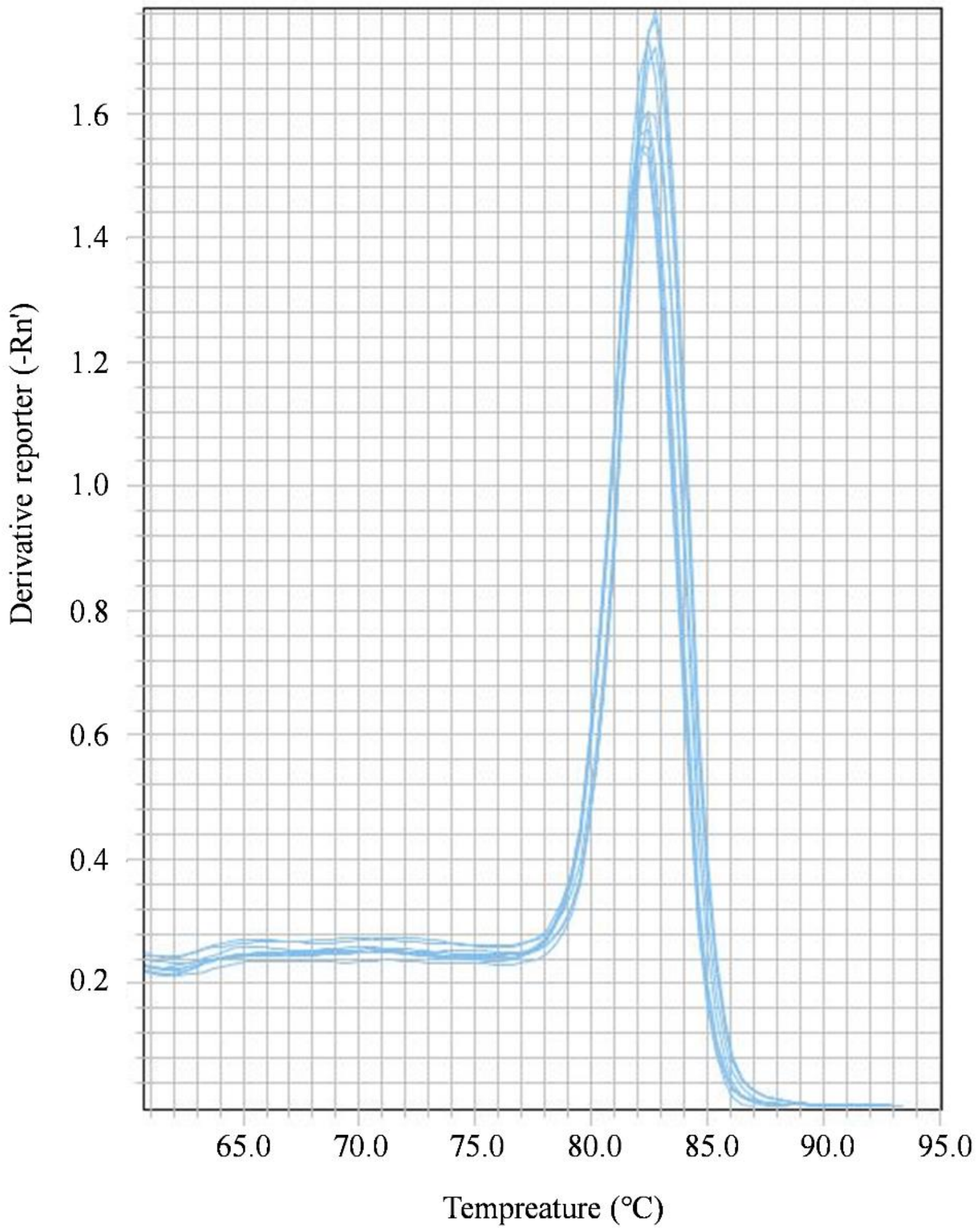

Fig. S17 BcBOA11 melt curve

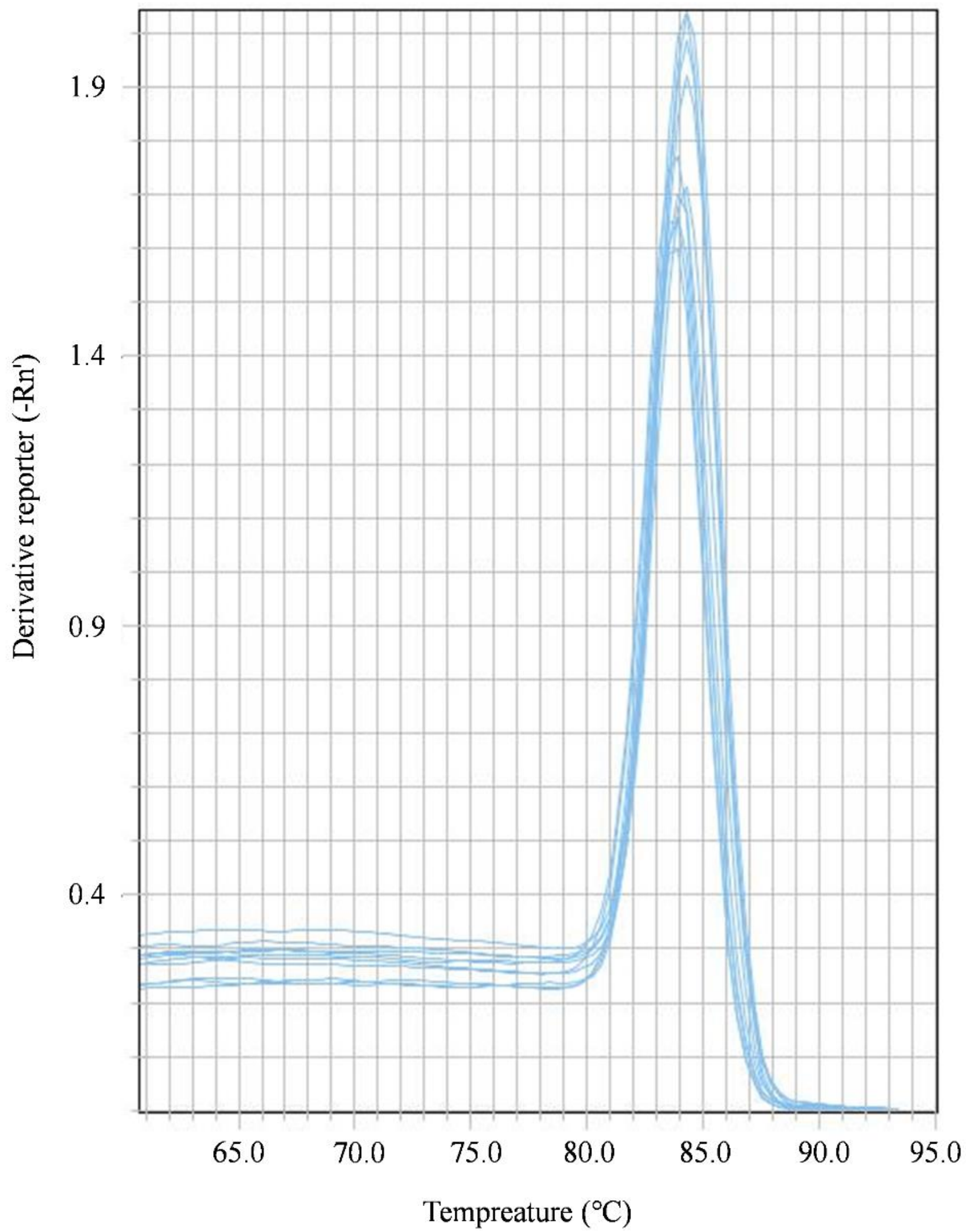

Fig. S18 Bcnob1 melt curve

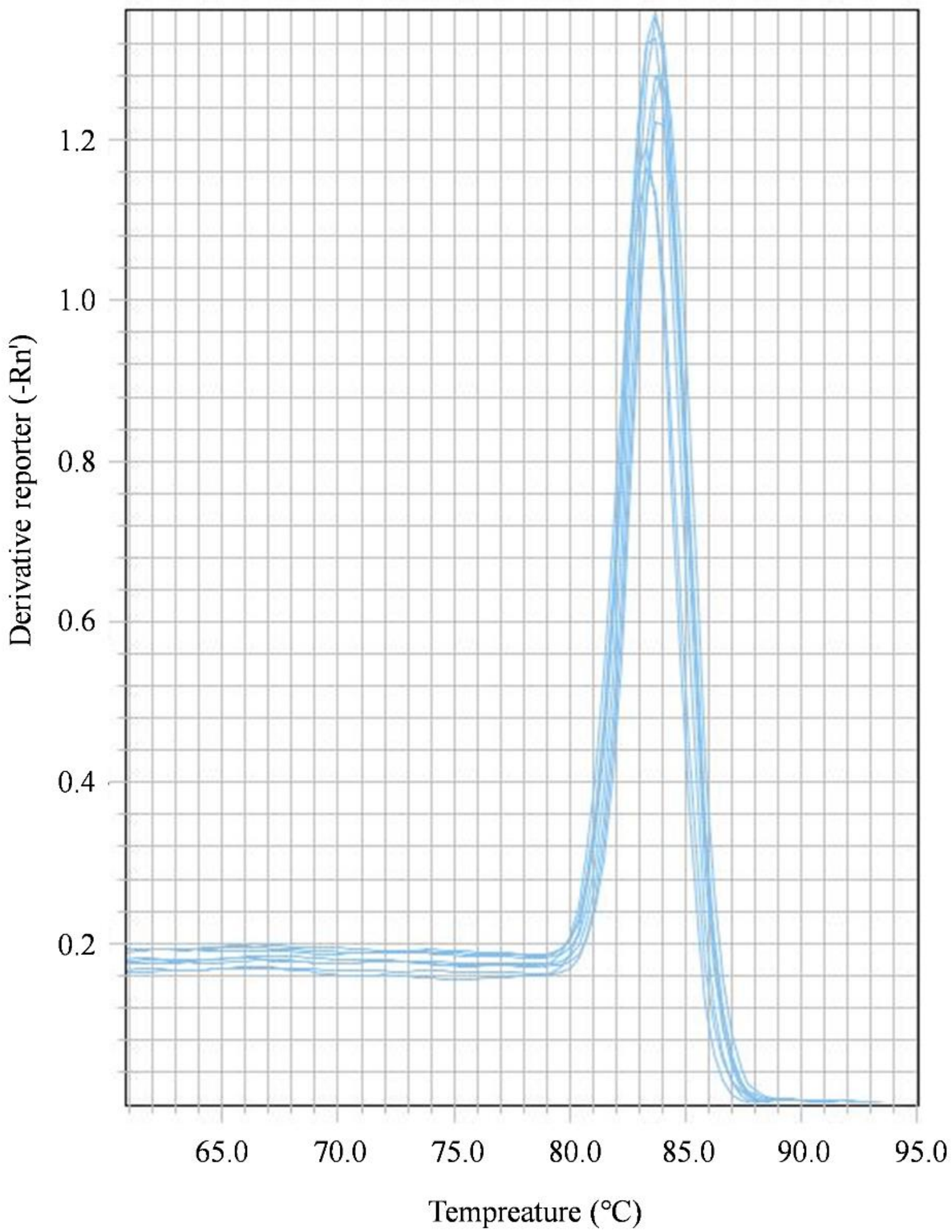

Fig. S19 Bcactin melt curve

Table S1. Oligonucleotide primers used in this study

Primer	Sequence(5’-3’)	Relevant characteristics
P1	GCTTGGTGACTTGAGGATGTTCT	PCR primers to amplify *Bcsdr2* upstream fragment for construction of the gene deletion vector
P2	ATATCATCTTCTGTCGACCTGCAGGCTGGAGGAGGAATGACTCATTCG	
P3	TCTTTCTAGAGGATCCCCGGGTACCGACTATTATCGCTGTATGGACTGA	PCR primers to amplify *Bcsdr2* downstream fragment for construction of the gene deletion vector
P4	ATCACGGCTTGTAACAATCAAG	
P5	GCTTGGTGACTTGAGGATGTTCT	PCR primers to amplify the deletion vector of *Bcsdr2* with the double-joint PCR products as template
P6	ATCACGGCTTGTAACAATCAAG	
P7	CGGTACCCGGGGATCCTCTAG	PCR primers to amplify the hygromycin B (*HPH*) gene
P8	GCCTGCAGGTCGACAGAAGATG	
P9	CGAGCCGAGTTCTGACGC	PCR primers to identificate the *Bcsdr2* deletion mutant
P10	CGACAAGTTTGGACAAGATTATG	
P11	TTTATCGTTACTGGGGTTTTGC	PCR primers to amplify the probe fragment for *Bcsdr2* Southern blotting
P12	GGACTTCTGGAGGAGGAATGAC	

Table S2 The mapping statistics in transcriptome analysis

Sample name	Clean reads	Total mapped	Multiple mapped	Uniquely mapped	Read-1
B05.10_1	46613948	45917632 (98.51%)	141044 (0.3%)	45776588 (98.2%)	22888294 (49.1%)
B05.10_2	39397118	38670088 (98.15%)	119836 (0.3%)	38550252 (97.85%)	19275126 (48.93%)
B05.10_3	44213524	43548774 (98.5%)	121226 (0.27%)	43427548 (98.22%)	21713774 (49.11%)
Bcest_1	45469618	44694020 (98.29%)	138078 (0.3%)	44555942 (97.99%)	22277971 (49%)
Bcest_2	40346458	39775210 (98.58%)	124850 (0.31%)	39650360 (98.27%)	19825180 (49.14%)
Bcest_3	43932620	43061752 (98.02%)	129820 (0.3%)	42931932 (97.72%)	21465966 (48.86%)
Sample name	Read-2	Reads map to '+'	Reads map to '-'	Non-splice reads	Splice reads
B05.10_1	22888294 (49.1%)	22888294 (49.1%)	22888294 (49.1%)	35537804 (76.24%)	10238784 (21.97%)
B05.10_2	19275126 (48.93%)	19275126 (48.93%)	19275126 (48.93%)	30056948 (76.29%)	8493304 (21.56%)
B05.10_3	21713774 (49.11%)	21713774 (49.11%)	21713774 (49.11%)	33180604 (75.05%)	10246944 (23.18%)
Bcest_1	22277971 (49%)	22277971 (49%)	22277971 (49%)	35186161 (77.38%)	9369781 (20.61%)
Bcest_2	19825180 (49.14%)	19825180 (49.14%)	19825180 (49.14%)	30509267 (75.62%)	9141093 (22.66%)
Bcest_3	21465966 (48.86%)	21465966 (48.86%)	21465966 (48.86%)	33533174 (76.33%)	9398758 (21.39%)

Table S3 List of all DEGs in transcriptome analyses

Gene_id	Readcount Bcsdr2	Readcount B05_10	log2Fold Change	*p* val	*p* adj	Start	End	Length	Description
Novel00195	116.3342899	45.67808443	1.3487	0.0015188	0.047037	1474733	1478456	3671	Hypothetical protein
Novel00354	171.2648814	69.77775722	1.2954	0.00066532	0.027403	941542	944392	2391	Predicted protein
Novel00437	142.2009823	351.6565423	-1.3062	0.00042162	0.020762	2022493	2027369	4738	Hypothetical protein
Novel00626	25.59960484	4.039118071	2.664	0.00042179	0.020762	264980	269120	4074	Hypothetical protein
gene-BCIN_01g00050	1115.129373	263.9585714	2.0788	0.00085845	0.032383	14259	15636	1378	Oxidoreductase
gene-BCIN_01g00100	965.7839339	211.330026	2.1922	3.65E-07	0.0001006	53107	54450	1165	BcBOA10 protein
gene-BCIN_01g00110	890.8758256	177.0901426	2.3307	0.00016728	0.01097	54595	56431	1837	BcBOA11 protein
gene-BCIN_01g00120	1350.717367	327.6034671	2.0437	0.0003624	0.018834	56498	58186	1689	Putative trichothecene 3-*O* -acetyltransferase protein
gene-BCIN_01g01320	1716.083827	463.7849666	1.8876	3.13E-09	1.65E-06	527881	530356	2244	Hypothetical protein
gene-BCIN_01g01570	836.1698739	3000.812311	-1.8435	2.22E-05	0.0024783	608012	610228	2164	Hypothetical protein
gene-BCIN_01g01590	1656.398221	16709.90658	-3.3346	7.36E-08	2.55E-05	621319	623189	1450	Hypothetical protein
gene-BCIN_01g01620	430.5120839	1786.411242	-2.0529	1.40E-09	8.48E-07	629357	631884	2123	Predicted protein
gene-BCIN_01g01630	675.8369553	1558.307998	-1.2052	0.00038326	0.019694	632040	634252	1919	Similar to cytochrome P450 monooxygenase
gene-BCIN_01g01840	1498.374275	3303.735558	-1.1407	0.00019651	0.012018	710632	711916	1157	Hypothetical protein
gene-BCIN_01g03400	2567.658298	1047.329164	1.2937	0.00016588	0.01097	1210085	1212110	1915	Similar to MFS multidrug transporter
gene-BCIN_01g03510	45.35595438	167.9504645	-1.8887	0.0014064	0.04451	1253430	1255741	2160	Similar to cytochrome P450 monooxygenase

gene-BCIN_01g03900	33.97307391	85.40552706	-1.3299	0.0013017	0.042312	1438919	1439531	439	Hypothetical protein
gene-BCIN_01g04090	1059.684745	2086.692699	-0.97758	0.0014845	0.046212	1495249	1496662	1289	Bcpno1
gene-BCIN_01g04230	3650.454168	1007.411849	1.8574	0.00063282	0.026333	1541543	1543226	1684	Bcmet16
gene-BCIN_01g04710	1616.000892	4444.77455	-1.4597	1.09E-05	0.0014546	1723098	1725511	2284	Bcnop7
gene-BCIN_01g04890	1141.602834	3887.479338	-1.7678	2.05E-05	0.0023476	1764882	1766899	1914	Putative cytochrome P450 protein
gene-BCIN_01g04900	1068.37964	3868.733006	-1.8564	0.00094885	0.034923	1768012	1769216	992	Putative glutathione *S* -transferase protein
gene-BCIN_01g05660	107.6125256	39.52282794	1.4451	0.00053869	0.02407	1967320	1968067	523	Hypothetical protein
gene-BCIN_01g05830	1270.208241	617.3608094	1.0409	0.00096802	0.035413	2037133	2039571	2251	Hypothetical protein
gene-BCIN_01g05840	459.6302988	212.2172536	1.1149	0.00059448	0.02517	2040442	2041176	735	Similar to prolyl oligopeptidase
gene-BCIN_01g05850	1075.404353	509.5182189	1.0777	0.00095529	0.035053	2041778	2045094	2966	Putative C2H2 type zinc finger domain protein
gene-BCIN_01g06660	11.5124074	47.00646748	-2.0297	0.00049415	0.022829	2336338	2337665	1328	Hypothetical protein
gene-BCIN_01g06930	3546.384099	7160.42832	-1.0137	0.0011643	0.039164	2427311	2429022	1608	BccarA
gene-BCIN_01g06990	472.6309182	1548.036511	-1.7117	1.03E-07	3.48E-05	2443737	2447029	3232	Hypothetical protein
gene-BCIN_01g07370	30.97986116	84.6444002	-1.4501	0.0008972	0.033352	2573169	2575146	1936	Hypothetical protein
gene-BCIN_01g07380	1966.950321	6061.2693	-1.6237	1.87E-05	0.0022206	2579515	2581666	2152	Hypothetical protein
gene-BCIN_01g07420	960.2956519	1997.67565	-1.0568	0.0010173	0.036019	2598233	2599920	1547	Bcpxr1
gene-BCIN_01g07450	514.2177578	1115.696553	-1.1175	0.00036184	0.018834	2602203	2603445	1172	Bcbud23
gene-BCIN_01g08120	22.48990308	78.2617963	-1.799	0.00011753	0.0085217	2843349	2845002	1654	Putative spherulin 4-like cell surface protein

gene-BCIN_01g09520	1955.521737	5029.618183	-1.3629	1.02E-05	0.0013717	3302622	3304330	1527	Putative upf0655 protein ycr015c protein
gene-BCIN_01g09590	2856.302003	5650.346661	-0.98419	0.0015147	0.047028	3333724	3336840	2583	Hypothetical protein
gene-BCIN_01g09630	339.9632655	4111.734703	-3.5963	0.00040511	0.020355	3343227	3344303	838	Hypothetical protein
gene-BCIN_01g09720	1356.172449	4272.178679	-1.6554	1.25E-07	4.08E-05	3378234	3381805	3277	Hypothetical protein
gene-BCIN_01g09780	8787.741074	4123.824094	1.0915	0.0010987	0.03801	3399995	3405731	4762	Similar to PH domain-containing protein
gene-BCIN_01g09970	851.9719374	2464.536418	-1.5324	0.00055267	0.024247	3478000	3480112	2059	Carbohydrate esterase family 9 protein
gene-BCIN_01g10130	503.0690406	10599.59068	-4.3971	3.48E-05	0.0033484	3535852	3538316	1865	Bcdug2
gene-BCIN_01g10140	311.993084	3972.783506	-3.6706	0.00012888	0.0090733	3539170	3540577	1348	Hypothetical protein
gene-BCIN_01g10150	3800.977209	46735.20127	-3.6201	0.00014708	0.010235	3542257	3543894	1638	Bcpsd
gene-BCIN_01g10480	649.425232	1363.260431	-1.0698	0.00062755	0.026267	3653256	3654388	1029	Bcrrs1
gene-BCIN_01g10810	14576.24754	33225.42841	-1.1887	0.00010959	0.0081916	3758683	3760798	2056	Putative cross-pathway control 1 protein
gene-BCIN_01g10840	2267.867637	8388.272689	-1.887	3.93E-05	0.0036094	3778305	3780521	1995	Hypothetical protein
gene-BCIN_01g11300	1305.105158	2898.100297	-1.1509	0.00022789	0.013729	3952662	3954596	1880	Hypothetical protein
gene-BCIN_02g00210	1338.920256	564.4521703	1.2461	8.58E-05	0.0067453	182943	186304	3116	Putative lipase 2 protein
gene-BCIN_02g00230	724.3036932	1968.712614	-1.4426	3.64E-06	0.0006681	192877	194449	1451	Similar to aldo/keto reductase family oxidoreductase
gene-BCIN_02g00240	1301.888348	4665.613085	-1.8415	0.0005915	0.025131	194806	197042	1947	Similar to cytochrome P450 monooxygenase
gene-BCIN_02g00300	339.4912751	1313.171031	-1.9516	0.00043057	0.021023	212360	214463	1709	Putative cyclopropane-fatty-acyl-phospholipid synthase protein
gene-BCIN_02g00360	4.506253736	106.9943608	-4.5695	4.09E-09	1.98E-06	233075	234361	1228	Putative nitrilase protein

gene-BCIN_02g00460	3717.002614	7673.573838	-1.0458	0.00080874	0.031343	256676	259090	2273	Hypothetical protein
gene-BCIN_02g00620	31.32043665	121.9040337	-1.9606	4.17E-06	0.0007007	311304	314139	2098	Putative MFS lactose protein
gene-BCIN_02g00630	2251.090237	4636.972713	-1.0426	0.00072177	0.02875	314457	317111	2389	Putative tRNA-splicing endonuclease subunit protein
gene-BCIN_02g01090	223.9799366	583.8010179	-1.3821	3.33E-05	0.0032286	466748	467858	1035	Hypothetical protein
gene-BCIN_02g01610	1247.050618	2592.199049	-1.0557	0.0016521	0.04936	633015	635194	2097	Hypothetical protein
gene-BCIN_02g01730	212.516755	1227.821407	-2.5305	1.07E-09	7.64E-07	670936	672772	1344	Putative zinc-binding oxidoreductase protein
gene-BCIN_02g01820	1501.150136	3086.959517	-1.0401	0.00090067	0.033352	701401	703981	2530	Bcort1
gene-BCIN_02g01830	579.369046	1415.979858	-1.2892	0.00015729	0.0107	704091	705248	1038	Bccgr1
gene-BCIN_02g03350	1418.804869	437.2350119	1.6982	0.0010633	0.037106	1212714	1213335	521	Bccox17
gene-BCIN_02g04360	338.6133595	1228.595481	-1.8593	0.00044795	0.021462	1549317	1551130	1552	Putative pigment biosynthesis protein ayg1 protein
gene-BCIN_02g04940	130.5931889	48.18391457	1.4385	0.00029037	0.016129	1766087	1767463	1377	Hypothetical protein
gene-BCIN_02g05720	334.4139547	155.0276296	1.1091	0.0010698	0.037226	2051728	2054472	2745	Putative heterokaryon incompatibility protein
gene-BCIN_02g06280	587.4378175	1633.810864	-1.4757	0.00018203	0.011544	2214031	2214903	820	Hypothetical protein
gene-BCIN_02g06300	307.6612816	1415.821513	-2.2022	8.99E-06	0.0012231	2217312	2219509	1896	Hypothetical protein
gene-BCIN_02g06310	92.12004648	447.6589949	-2.2808	2.02E-05	0.0023475	2219673	2221608	1878	Similar to delta(12) fatty acid desaturase
gene-BCIN_02g06320	391.028651	1414.192837	-1.8546	1.15E-08	4.79E-06	2223183	2225576	2158	Similar to MFS multidrug transporter
gene-BCIN_02g06330	56.50287012	246.3040716	-2.124	1.30E-07	4.15E-05	2225585	2227549	1866	Hypothetical protein
gene-BCIN_02g06340	92.93009872	534.4211682	-2.5238	2.10E-09	1.21E-06	2228343	2229971	1459	Bcprd6

gene-BCIN_02g06350	702.1545194	2179.936554	-1.6344	2.40E-07	7.27E-05	2230123	2231622	1321	Similar to hemolysin-III channel protein Izh2
gene-BCIN_02g06490	687.2952622	1409.363049	-1.036	0.0011283	0.038595	2268358	2269977	1525	Bctsr4
gene-BCIN_02g06690	566.1969056	275.4806186	1.0394	0.0012985	0.042312	2343905	2346098	2029	Similar to cytochrome P450 alkane hydroxylase
gene-BCIN_02g07100	694.1728059	215.5944997	1.687	0.00036096	0.018834	2511449	2512622	958	Hypothetical protein
gene-BCIN_02g07280	177.6099755	396.4899136	-1.1586	0.00047311	0.022119	2577135	2578410	1173	Hypothetical protein
gene-BCIN_02g07390	310.7513853	681.389086	-1.1327	0.0005074	0.023273	2617467	2622153	4518	Hypothetical protein
gene-BCIN_02g07470	142.5260672	32.54816284	2.1306	0.00055667	0.024335	2655866	2657037	1105	Hypothetical protein
gene-BCIN_02g07490	57.3688604	18.23008186	1.6539	0.0013355	0.043124	2659748	2661031	1103	Hypothetical protein
gene-BCIN_02g07630	3286.705515	11836.78399	-1.8486	0.00061059	0.025762	2712808	2714969	1817	Hypothetical protein
gene-BCIN_02g07640	2497.636044	9982.916247	-1.9989	0.0013703	0.044014	2716773	2719282	2395	Bclcc7
gene-BCIN_02g07820	18.92905099	83.16178628	-2.1353	3.55E-06	0.0006606	2824211	2825496	1214	Putative prolyl aminopeptidase protein
gene-BCIN_02g08110	459.4360526	1172.617624	-1.3518	3.09E-05	0.0030927	2899844	2901712	1787	Bcutp6
gene-BCIN_02g08540	2957.956127	5944.975133	-1.0071	0.0010549	0.03692	3036562	3038722	2052	Similar to pre-rRNA processing nucleolar protein Sik1
gene-BCIN_02g08610	714.871221	1484.795595	-1.0545	0.0015395	0.047554	3056175	3058321	1991	Hypothetical protein
gene-BCIN_02g08900	43.01140643	115.393875	-1.4238	0.00034471	0.018388	3168276	3169704	1269	Putative Tat pathway signal sequence
gene-BCIN_03g00060	129.12669	1156.863265	-3.1634	0.0014378	0.044989	33773	35618	1723	Hypothetical protein
gene-BCIN_03g00070	46.00331793	164.3855631	-1.8373	7.88E-06	0.001148	36270	38123	1682	Similar to MFS transporter
gene-BCIN_03g00080	45.4975308	143.2089952	-1.6543	0.0011472	0.038802	38780	40045	1204	Putative taurine catabolism dioxygenase protein

gene-BCIN_03g00530	176.7608891	392.3880218	-1.1505	0.0005458	0.024141	194751	196495	1745	Hypothetical protein
gene-BCIN_03g02100	166.2157629	438.0692696	-1.3981	3.81E-05	0.00355	701997	703210	994	Similar to ribonuclease p/mrp subunit
gene-BCIN_03g02570	1128.08942	3033.042775	-1.4269	1.82E-05	0.0022039	847390	848598	1097	Hypothetical protein
gene-BCIN_03g02860	19970.95827	44134.16595	-1.144	0.00017752	0.011431	948597	951292	2232	Similar to psi protein
gene-BCIN_03g02870	748.4431783	1670.658837	-1.1585	0.00026719	0.01519	952059	953195	1023	Hypothetical protein
gene-BCIN_03g02950	648.5974494	1392.237761	-1.102	0.00049583	0.022829	974359	975931	1573	Bcrpf1
gene-BCIN_03g03230	711.4128194	1707.399247	-1.263	0.00070682	0.02853	1075514	1077692	2129	Similar to Sas10/Utp3 family protein
gene-BCIN_03g03410	195.1763444	84.16943993	1.2134	0.00081051	0.031343	1139848	1149349	8961	Putative toxin subunit protein
gene-BCIN_03g04900	1592.292305	3202.033743	-1.0079	0.0011887	0.039654	1656217	1658205	1989	Putative srp40-c-like protein
gene-BCIN_03g04920	522.6506929	186.2096944	1.4889	8.29E-05	0.0066068	1663617	1665525	1787	Pimilar to MFS monocarboxylate transporter
gene-BCIN_03g05160	77.50461877	18.82865355	2.0414	1.63E-05	0.0020166	1745254	1746706	1244	Hypothetical protein
gene-BCIN_03g05220	985.7608604	2167.994492	-1.1371	0.00099928	0.036019	1762243	1765319	3020	Hypothetical protein
gene-BCIN_03g05230	670.2530597	129.0135758	2.3772	4.05E-06	0.0007	1765889	1768601	2390	Hypothetical protein
gene-BCIN_03g05720	909.1053083	2183.114904	-1.2639	0.00081277	0.031343	1916045	1916967	860	Hypothetical protein
gene-BCIN_03g05760	163.6419937	63.8773677	1.3572	0.00035258	0.0187	1936661	1937730	1070	Similar to tricarboxylate transport protein (mitochondrial)
gene-BCIN_03g05790	1732.526711	3772.281982	-1.1226	0.00028984	0.016129	1943354	1945031	1544	Bcrpa49
gene-BCIN_03g06050	133.622116	289.2835413	-1.1143	0.001284	0.04225	2036359	2037436	864	Bcfap7
gene-BCIN_03g06190	4076.343746	9982.189496	-1.2921	1.95E-05	0.0022912	2073642	2075278	1495	Hypothetical protein

gene-BCIN_03g06340	1600.925509	3947.537801	-1.302	0.001136	0.038647	2117647	2119891	1939	Hypothetical protein
gene-BCIN_03g06600	229422.3867	469383.4485	-1.0328	0.00081072	0.031343	2228667	2231555	2336	Similar to heat shock protein 70
gene-BCIN_03g06610	649.7529129	2039.818683	-1.6505	2.58E-07	7.43E-05	2234502	2236593	2092	Hypothetical protein
gene-BCIN_03g07210	1689.086997	5035.684519	-1.5759	2.47E-07	7.31E-05	2443260	2445477	1552	Similar to prolyl oligopeptidase
gene-BCIN_03g07930	1439.232231	2962.430153	-1.0415	0.00082324	0.031447	2723387	2725751	2210	Putative RNA-binding la domain-containing protein
gene-BCIN_03g08100	988.2112507	2804.818699	-1.505	2.39E-06	0.0004585	2787239	2788499	1160	Similar to 1,3,8-naphthalenetriol reductase
gene-BCIN_03g08530	383.5520711	1177.829405	-1.6186	6.23E-05	0.0052407	2916915	2920202	3083	Hypothetical protein
gene-BCIN_03g08710	156.9299327	386.5257643	-1.3004	0.00019451	0.011956	2980302	2983573	2799	Glycoside hydrolase family 3 protein
gene-BCIN_03g08920	4950.313701	436.8092496	3.5024	9.52E-09	4.12E-06	3049527	3052148	2570	Putative MFS transporter protein
gene-BCIN_03g09050	617.3702727	1390.056036	-1.1709	0.00030964	0.016848	3094975	3097177	2136	Hypothetical protein
gene-BCIN_03g09200	119.6043489	275.3172928	-1.2028	0.00058185	0.024896	3163790	3165458	1460	Hutative P-type atpase protein
gene-BCIN_04g00080	242.9243058	510.8375859	-1.0724	0.00068294	0.027844	37945	39317	1373	Hypothetical protein
gene-BCIN_04g00590	235.4825046	488.742202	-1.0535	0.0012559	0.041535	222686	224729	1900	hypothetical protein
gene-BCIN_04g00900	1785.782404	3672.630769	-1.0403	0.00045485	0.021597	333181	339410	5956	Putative ankyrin repeat-containing protein
gene-BCIN_04g01020	970.3708231	2141.747739	-1.1422	0.00023985	0.014237	403358	407303	3831	Putative vegetative incompatibility protein het-e-1 protein
gene-BCIN_04g01520	2621.806684	7884.656624	-1.5885	8.55E-07	0.0001992	569369	571677	1940	Hypothetical protein
gene-BCIN_04g03020	1018.320933	2401.899998	-1.238	9.09E-05	0.0070989	1082198	1083912	1665	Putative ribosomal biogenesis protein gar2 protein
gene-BCIN_04g03310	800.0991977	349.1957401	1.1961	0.00055253	0.024247	1195887	1197418	1348	Hypothetical protein

gene-BCIN_04g03400	818.5532333	1992.688894	-1.2836	0.00024164	0.014268	1219968	1222430	2463	Hypothetical protein
gene-BCIN_04g03750	325.2896008	665.5062906	-1.0327	0.0013036	0.042312	1344128	1346957	2646	Similar to amino acid transporter
gene-BCIN_04g04150	14848.03638	3849.128338	1.9477	8.06E-10	6.10E-07	1473160	1474205	937	Hypothetical protein
gene-BCIN_04g05000	424.511133	2428.620552	-2.5163	8.29E-05	0.0066068	1724037	1728750	4123	Similar to fatty acid oxygenase
gene-BCIN_04g05960	166.0039592	501.6316318	-1.5954	8.77E-06	0.0012065	2046041	2047794	1641	Hypothetical protein
gene-BCIN_04g06450	7454.887916	22942.01593	-1.6217	0.00067259	0.027608	2222160	2223749	1275	Hypothetical protein
gene-BCIN_04g06650	19833.08351	42502.81202	-1.0996	0.0013069	0.042312	2303388	2306101	2605	Hypothetical protein
gene-BCIN_05g00070	947.1247786	2074.017122	-1.1308	0.00062906	0.026267	45545	47574	2030	Bcrrp9
gene-BCIN_05g00320	529.7767415	1285.662168	-1.2791	6.43E-05	0.0052951	135554	137475	1922	Hypothetical protein
gene-BCIN_05g01220	277.5993626	581.9852324	-1.068	0.0013959	0.04451	489127	493116	3939	Hypothetical protein
gene-BCIN_05g01430	572.3951643	1318.227556	-1.2035	0.00019153	0.01188	568584	571109	2142	Hypothetical protein
gene-BCIN_05g01450	82.18938496	23.63267856	1.7982	9.95E-05	0.0076723	573177	575869	2646	Bcprd2
gene-BCIN_05g01460	162.5669532	64.91601495	1.3244	0.00057951	0.024884	576671	579143	1812	Hypothetical protein
gene-BCIN_05g01520	1731.314971	4645.258824	-1.4239	4.00E-06	0.0007	589000	590781	1731	Hypothetical protein
gene-BCIN_05g01530	2155.184449	7601.992537	-1.8186	5.54E-05	0.0047551	592858	596194	3084	Hypothetical protein
gene-BCIN_05g01980	406.9101004	937.3320062	-1.2039	8.05E-05	0.0065405	743872	746024	2153	Hypothetical protein
gene-BCIN_05g02490	821.518676	283.5832063	1.5345	2.32E-06	0.0004526	914078	915225	1094	Bcmxr1
gene-BCIN_05g02820	191.4910705	435.5048358	-1.1854	0.00039413	0.019969	1027849	1030600	2699	Hypothetical protein

gene-BCIN_05g04130	440.7132381	177.3098679	1.3136	0.001628	0.048988	1484983	1485784	802	Hypothetical protein
gene-BCIN_05g04400	157.1574603	518.1680744	-1.7212	1.78E-05	0.0021748	1566551	1567478	875	Hypothetical protein
gene-BCIN_05g04840	90.41880458	268.1692967	-1.5684	0.000409	0.020381	1711741	1714243	2346	Similar to urea active transporter
gene-BCIN_05g04990	68.62274793	168.0657722	-1.2923	0.00052934	0.023917	1777455	1779439	1985	Similar to short-chain dehydrogenase
gene-BCIN_05g05000	56.00335991	154.473074	-1.4638	0.00010128	0.0077618	1779629	1781132	1504	Hypothetical protein
gene-BCIN_05g05120	399.2584439	1618.647463	-2.0194	0.00011234	0.0082443	1820340	1821885	1357	Similar to short chain dehydrogenase
gene-BCIN_05g05480	424.1759499	869.2518746	-1.0351	0.0014053	0.04451	1925314	1927564	2251	Similar to arrestin (or S-antigen) domain protein
gene-BCIN_05g05490	476.7677661	226.3508426	1.0747	0.0012588	0.041535	1929590	1932695	3106	Hypothetical protein
gene-BCIN_05g05620	567.9543873	1262.382091	-1.1523	0.00025751	0.014836	1975206	1977259	2001	Putative pseudouridylate synthase 3 protein
gene-BCIN_05g05790	2390.336709	4957.873801	-1.0525	0.00077348	0.03041	2007064	2009319	2128	Bcnop58
gene-BCIN_05g06180	451.4022693	968.5047621	-1.1013	0.00052568	0.023841	2115121	2117927	2664	Hypothetical protein
gene-BCIN_05g06490	97.0736142	452.1049227	-2.2195	8.06E-11	6.97E-08	2228508	2229403	840	Hypothetical protein
gene-BCIN_05g06500	99.96966491	703.9966235	-2.816	7.65E-16	1.85E-12	2229989	2232387	2340	Hypothetical protein
gene-BCIN_05g07090	433.3432551	1355.624507	-1.6454	0.00025745	0.014836	2464985	2467413	2209	Similar to cytochrome P450 monooxygenase
gene-BCIN_05g07640	652.3889105	5386.621956	-3.0456	1.12E-11	1.13E-08	2703252	2705977	2113	Putative elymoclavine monooxygenase protein
gene-BCIN_05g08280	4103.713696	10117.24942	-1.3018	2.64E-05	0.0027829	2888840	2892641	3550	Hypothetical protein
gene-BCIN_06g00730	1114.023207	2302.845118	-1.0476	0.00093426	0.034491	298647	300426	1731	Similar to rRNA processing protein Bystin
gene-BCIN_06g00790	877.0565837	1756.484978	-1.0019	0.0016461	0.049339	313265	315201	1827	Similar to ribose-phosphate pyrophosphokinase

gene-BCIN_06g00970	133.4689324	425.0522789	-1.6711	0.00012021	0.0086134	364009	365340	1332	Hypothetical protein
gene-BCIN_06g01220	77.890001	312.8849202	-2.0061	3.86E-08	1.42E-05	444005	446681	2620	Putative heterokaryon incompatibility protein
gene-BCIN_06g01460	110.0247981	343.7044228	-1.6433	0.0010087	0.036019	519380	523231	3852	Hypothetical protein
gene-BCIN_06g02020	321.4861739	951.0552844	-1.5648	1.20E-06	0.0002684	714987	717031	1923	Bcap11
gene-BCIN_06g02030	545.6143023	1478.572662	-1.4383	4.47E-06	0.0007411	717364	719282	1516	Hypothetical protein
gene-BCIN_06g02350	3019.162337	6962.803528	-1.2055	0.0001106	0.0082151	819760	822221	1990	Bcnog2
gene-BCIN_06g02670	19.44285042	120.2208436	-2.6284	2.37E-09	1.31E-06	915047	916867	1714	Putative cytochrome P450 alkane hydroxylase protein
gene-BCIN_06g02680	25.82104706	147.985147	-2.5188	3.48E-09	1.76E-06	917528	918641	1012	Hypothetical protein
gene-BCIN_06g03290	663.779838	1460.213963	-1.1374	0.00031634	0.017025	1149963	1151839	1829	Bcpwp1
gene-BCIN_06g03430	339.1382513	154.686446	1.1325	0.0012382	0.041191	1197042	1200812	3608	Hypothetical protein
gene-BCIN_06g03510	4162.070886	8275.515311	-0.99155	0.0010388	0.036462	1229538	1232182	1863	Putative transcription factor sequence protein
gene-BCIN_06g03630	788.3014951	287.7222486	1.4541	0.00012424	0.0088496	1272193	1274868	2477	Similar to transcription factor Cys6
gene-BCIN_06g03960	78.82914602	223.9878191	-1.5066	4.56E-05	0.0040594	1361898	1364264	2232	Hypothetical protein
gene-BCIN_06g04140	593.1507874	1780.024487	-1.5854	6.40E-05	0.0052951	1430911	1432920	1811	Hypothetical protein
gene-BCIN_06g04170	78.58622878	192.7251886	-1.2942	0.00058461	0.024926	1438772	1441386	2476	Putative heterokaryon incompatibility protein
gene-BCIN_06g04860	148.1033336	1477.008282	-3.318	5.51E-22	2.22E-18	1649541	1652033	1836	Similar to MFS nicotinic acid transporter
gene-BCIN_06g04870	50.96151888	164.2203615	-1.6882	0.0014126	0.044546	1653014	1655268	2079	Similar to G-protein coupled receptor
gene-BCIN_06g05570	2661.771248	5913.090945	-1.1515	0.00018963	0.011845	1905931	1907341	1411	Hypothetical protein

gene-BCIN_06g05640	997.2402477	2156.263067	-1.1125	0.00045786	0.021597	1941860	1943279	1369	Bcrpc40
gene-BCIN_06g05890	1073.946215	536.8390772	1.0004	0.0010054	0.036019	2051433	2054903	3367	Hypothetical protein
gene-BCIN_06g06280	47186.14839	92793.05163	-0.97565	0.0013012	0.042312	2181941	2184665	2225	Similar to mitochondrial protein import protein MAS5
gene-BCIN_06g06690	605.9531081	1374.689932	-1.1818	0.00070217	0.028437	2340715	2342586	1809	Putative microtubule associated protein
gene-BCIN_06g06730	77.02091202	176.896167	-1.1996	0.0011394	0.038647	2358688	2360097	1410	Hypothetical protein
gene-BCIN_06g06810	371.1266862	746.3192684	-1.0079	0.0014078	0.04451	2383047	2384246	1066	Bcfcf1
gene-BCIN_06g06940	671.8190816	1353.900821	-1.011	0.00099825	0.036019	2425585	2426691	965	Similar to adoMet-dependent tRNA methyltransferase
gene-BCIN_06g07160	91.81970657	229.5213812	-1.3218	0.00022502	0.013624	2537913	2539894	1773	hypothetical protein
gene-BCIN_06g07560	341.237286	799.6199856	-1.2285	0.00017404	0.011331	2665850	2667223	1374	Bclcp5
gene-BCIN_06g07580	14.87645023	49.70201407	-1.7403	0.00085603	0.032383	2670209	2672017	1647	Hypothetical protein
gene-BCIN_07g00070	280.8159323	781.611944	-1.4768	0.00038933	0.019808	35152	37487	2336	Hypothetical protein
gene-BCIN_07g00370	719.8159208	342.6379705	1.0709	0.0010223	0.036091	125948	128252	2305	Hypothetical protein
gene-BCIN_07g00550	336.1497217	130.4610577	1.3655	0.0015712	0.047925	209475	211784	1904	Hypothetical protein
gene-BCIN_07g00710	1754.423098	4775.490604	-1.4447	5.15E-06	0.0008203	251208	253649	2337	Hypothetical protein
gene-BCIN_07g01290	36169.33139	79440.32829	-1.1351	0.00021005	0.012782	467513	470720	2824	Putative heat shock protein Hsp88
gene-BCIN_07g01300	2126.120811	4638.316517	-1.1254	0.00043622	0.021213	471608	475862	3995	BcCHSVII, class VII chitin synthase
gene-BCIN_07g01470	88.84979593	237.6260349	-1.4193	0.00013596	0.0095165	536982	539148	2051	Hypothetical protein
gene-BCIN_07g01580	2365.661756	5898.415893	-1.3181	9.85E-05	0.0076441	587404	589997	2434	Similar to nucleolar GTP-binding protein

gene-BCIN_07g01660	391.4766231	167.3242464	1.2263	0.00035929	0.018834	613681	615258	1340	Hypothetical protein
gene-BCIN_07g02450	526.7594994	1128.369814	-1.099	0.00051683	0.023527	916611	918664	2004	Bcutp18
gene-BCIN_07g02890	24.10224601	120.0349753	-2.3162	0.00039669	0.020015	1050171	1051405	1196	Hypothetical protein
gene-BCIN_07g03410	155.5479625	368.6332033	-1.2448	0.00024566	0.014371	1254481	1256071	1591	Bccns1
gene-BCIN_07g03420	116.6185849	48.37712092	1.2694	0.001675	0.049834	1256136	1258681	2159	Similar to cation diffusion facilitator family metal ion transporter
gene-BCIN_07g03430	966.7878331	420.7320871	1.2003	0.00044841	0.021462	1258816	1260308	1376	Hypothetical protein
gene-BCIN_07g03950	727.1666716	1550.041693	-1.0919	0.00054427	0.024141	1429123	1431250	2027	RecName: Full=ATP-dependent RNA helicase dbp9
gene-BCIN_07g04770	147.772255	660.5298717	-2.1602	0.00043	0.021023	1728147	1730155	1958	Hypothetical protein
gene-BCIN_07g04810	1037.977913	2593.339058	-1.321	2.34E-05	0.0025538	1741785	1744326	2456	Hypothetical protein
gene-BCIN_07g05320	1619.258204	680.7744716	1.2501	3.77E-05	0.0035372	1969303	1971432	1970	Hypothetical protein
gene-BCIN_07g05330	884.9965392	338.3513808	1.3871	7.75E-06	0.0011454	1971885	1974253	2300	Hypothetical protein
gene-BCIN_07g05340	1186.421277	420.250821	1.4973	2.27E-05	0.0025015	1977489	1981521	3879	Hypothetical protein
gene-BCIN_07g05460	555.7574812	1158.785702	-1.0601	0.00068721	0.027924	2008797	2010513	1597	Hypothetical protein
gene-BCIN_07g05560	237.9403797	888.1185	-1.9002	7.72E-07	0.0001833	2054281	2056651	1519	Hypothetical protein
gene-BCIN_07g05890	2305.011366	5176.051107	-1.1671	0.00016508	0.01097	2175530	2177863	2012	Similar to CTP synthase
gene-BCIN_07g06040	49.21700478	14.70100377	1.7432	0.0015492	0.047613	2228101	2229884	1731	Hypothetical protein
gene-BCIN_07g06320	969.656678	473.1838751	1.0351	0.0010154	0.036019	2329188	2332172	2771	Similar to transcription factor Cys6
gene-BCIN_07g07110	1829.105573	3686.949486	-1.0113	0.0015563	0.047708	2629605	2632202	2483	Similar to amino acid transporter

gene-BCIN_08g00280	55.04369866	269.6408606	-2.2924	2.81E-05	0.0029314	116436	118298	1705	Similar to carboxypeptidase S1
gene-BCIN_08g00630	1094.891462	2743.886542	-1.3254	2.47E-05	0.0026432	278220	280018	1731	Similar to glutamate-rich WD repeat containing protein 1
gene-BCIN_08g00720	6894.816999	2859.656482	1.2697	0.00016128	0.010849	319398	320839	1389	Similar to pepsin
gene-BCIN_08g01060	77.6594043	183.3730869	-1.2395	0.0011394	0.038647	428085	429898	1741	Hypothetical protein
gene-BCIN_08g01290	134.7963372	381.783728	-1.502	0.00033003	0.017683	520510	522160	1651	Hypothetical protein
gene-BCIN_08g01780	933.4680011	2122.642414	-1.1852	0.00015587	0.010663	694552	696922	2220	Similar to MFS transporter
gene-BCIN_08g01790	1797.846384	3513.992604	-0.96684	0.0011092	0.038236	697294	699354	1944	Hypothetical protein
gene-BCIN_08g01960	1494.898417	3257.103029	-1.1235	0.00024273	0.014268	748386	750393	1854	Similar to RTA1 domain protein
gene-BCIN_08g01970	29.43016734	77.72842272	-1.4011	0.001655	0.04936	750754	753313	2472	Hypothetical protein
gene-BCIN_08g02070	449.1421879	1043.591203	-1.2163	0.00015178	0.010502	787623	790042	2110	Hypothetical protein
gene-BCIN_08g02740	1350.266549	643.5728294	1.0691	0.00086385	0.032486	1041950	1044363	2172	Similar to MFS multidrug transporter
gene-BCIN_08g03480	1128.423833	2731.638532	-1.2755	2.93E-05	0.0030088	1335227	1338190	2904	Hypothetical protein
gene-BCIN_08g04400	19.24928147	2.409590858	2.9979	0.0013605	0.043815	1720359	1721728	1322	Hypothetical protein
gene-BCIN_08g04600	1232.665242	580.784815	1.0857	0.001404	0.04451	1773522	1774654	1060	Similar to DSBA-like thioredoxin domain protein
gene-BCIN_08g04630	110.7858648	303.2408835	-1.4527	3.99E-05	0.0036324	1780554	1782020	1467	Hypothetical protein
gene-BCIN_08g05250	562.2858203	1389.693925	-1.3054	3.71E-05	0.003511	1981615	1983284	1341	Bcmrt4
gene-BCIN_08g05420	150.8127383	328.7203775	-1.1241	0.0010055	0.036019	2034644	2036725	2012	Hypothetical protein
gene-BCIN_08g05890	945.1547822	1960.960119	-1.0529	0.00071238	0.028564	2210726	2212378	1549	Putative rrna processing protein ebp2 protein

gene-BCIN_08g06240	202.2464281	667.4917364	-1.7226	3.33E-05	0.0032286	2324678	2327149	2096	Similar to salicylate hydroxylase
gene-BCIN_08g06270	631.5732447	254.0490727	1.3138	3.92E-05	0.0036094	2337435	2338987	1553	Hypothetical protein
gene-BCIN_08g06420	121.4974836	688.9952525	-2.5036	1.29E-13	1.96E-10	2370374	2372093	1408	Hypothetical protein
gene-BCIN_08g06800	532.2151142	161.119513	1.7239	4.93E-07	0.000127	2501452	2503743	2241	Hypothetical protein
gene-BCIN_08g06810	142.2031925	32.30543199	2.1381	7.76E-06	0.0011454	2503903	2505901	1999	Hypothetical protein
gene-BCIN_08g06820	65.00018523	18.50438666	1.8126	0.00031027	0.016848	2506006	2507987	1982	Putative iws1-like protein
gene-BCIN_08g07090	130.0941035	48.21128671	1.4321	0.00045838	0.021597	2593721	2594836	826	Putative glyoxylate reductase protein
gene-BCIN_09g00120	9247.612784	18829.96139	-1.0259	0.00057403	0.02484	39665	41048	1320	Bcget3
gene-BCIN_09g01390	836.2509521	1901.615765	-1.1852	0.00016759	0.01097	519280	521486	2207	Bcutp15
gene-BCIN_09g02160	415.3199865	168.3388323	1.3029	0.00010779	0.0081072	801793	803205	1413	Hypothetical protein
gene-BCIN_09g02170	580.9409821	1452.878258	-1.3225	3.02E-05	0.0030693	803467	804929	1405	Similar to dimethyladenosine transferase
gene-BCIN_09g02180	567.44735	1673.156555	-1.56	1.30E-06	0.0002853	805500	808335	2789	Bctsr1
gene-BCIN_09g02510	619.9992655	289.1894156	1.1003	0.00057678	0.024855	898408	908142	9030	Hypothetical protein
gene-BCIN_09g02520	52.15523828	13.97091808	1.9004	0.00053698	0.02407	908419	909373	891	Hypothetical protein
gene-BCIN_09g02640	320.7897157	113.8835465	1.4941	1.32E-05	0.0017032	951701	954861	2974	Hypothetical protein
gene-BCIN_09g03260	837.8792029	1986.464506	-1.2454	0.00011444	0.0083482	1162745	1167275	4467	Hypothetical protein
gene-BCIN_09g03650	52.82001936	173.6848612	-1.7173	0.0010322	0.036335	1304775	1306467	1645	Hypothetical protein
gene-BCIN_09g03660	47.41788349	179.0797662	-1.9171	0.00084988	0.032261	1307039	1308848	1558	Hypothetical protein

gene-BCIN_09g03670	1.53168024	42.28352505	-4.7869	1.27E-09	8.48E-07	1309104	1311334	1869	Similar to amino acid transporter
gene-BCIN_09g04390	146.8337345	1054.030399	-2.8437	1.89E-06	0.0003821	1540815	1541921	1107	Hypothetical protein
gene-BCIN_09g04490	1278.917836	570.5800547	1.1644	0.00075959	0.029961	1571012	1574573	3453	Glycoside hydrolase family 65 protein
gene-BCIN_09g04570	143.8697084	423.1165034	-1.5563	8.47E-06	0.0011928	1606037	1608597	2342	Putative choline dehydrogenase protein
gene-BCIN_09g04660	444.699361	1127.309618	-1.342	2.90E-05	0.0029979	1642009	1643286	1221	Hypothetical protein
gene-BCIN_09g04670	25.18462329	70.44215961	-1.4839	0.001591	0.048122	1643436	1645058	1473	Hypothetical protein
gene-BCIN_09g06320	108.1525424	24.01115695	2.1713	5.61E-05	0.0047868	2200928	2202737	1618	Hypothetical protein
gene-BCIN_09g06330	1425.043462	676.7749361	1.0743	0.00089525	0.033352	2202996	2205739	2616	Hypothetical protein
gene-BCIN_09g06730	207.8989798	1038.62559	-2.3207	0.0013744	0.044027	2377452	2379537	1918	Hypothetical protein
gene-BCIN_09g07130	19395.37916	40197.1457	-1.0514	0.00040865	0.020381	2539836	2541718	1631	Hypothetical protein
gene-BCIN_09g07140	8054.31954	19643.03318	-1.2862	2.06E-05	0.0023476	2544946	2546931	1579	Hypothetical protein
gene-BCIN_10g00010	4028.370839	10674.20751	-1.4059	7.60E-06	0.0011454	107	2148	1812	Hypothetical protein
gene-BCIN_10g00020	1147.662661	5100.098328	-2.1518	0.001244	0.04127	2889	5141	2187	Hypothetical protein
gene-BCIN_10g00300	211157.4741	434285.5325	-1.0403	0.00071888	0.028729	105547	108382	2579	Similar to heat shock protein 90
gene-BCIN_10g00500	124.4539019	397.0508749	-1.6737	4.04E-06	0.0007	185161	186816	1605	Hypothetical protein
gene-BCIN_10g00520	2097.834585	4997.36432	-1.2523	0.0003067	0.016848	194037	197526	3143	Putative zinc finger protein
gene-BCIN_10g01310	99.47259812	22.65541762	2.1344	1.78E-06	0.0003655	506094	508952	2565	Putative MFS multidrug protein
gene-BCIN_10g01340	147.7017556	30.25496101	2.2874	2.84E-08	1.11E-05	516656	518236	1581	Hypothetical protein

gene-BCIN_10g01350	1.965301042	156.9299917	-6.3192	5.13E-08	1.83E-05	518945	520449	1365	Similar to short-chain dehydrogenase/reductase SDR
gene-BCIN_10g01360	76.16295435	8.580294223	3.15	6.60E-07	0.00016	520458	521553	1096	Hypothetical protein
gene-BCIN_10g01370	144.6627651	45.84046046	1.658	6.31E-05	0.0052658	521616	527616	5891	Hypothetical protein
gene-BCIN_10g01500	8356.83691	3375.334853	1.3079	0.00027672	0.015585	573550	577578	3764	Similar to ATP synthase protein 9
gene-BCIN_10g01710	53.27210818	146.6148677	-1.4606	0.00018209	0.011544	664233	667647	3276	Hypothetical protein
gene-BCIN_10g02330	2122.767773	4538.012804	-1.0961	0.0011736	0.039258	900307	901665	1220	Bcrpo26
gene-BCIN_10g02660	10872.75115	31900.01078	-1.5528	3.05E-07	8.59E-05	1008927	1010368	1374	Hypothetical protein
gene-BCIN_10g02670	87.31640993	211.79575	-1.2783	0.00051605	0.023527	1010372	1011846	1475	Hypothetical protein
gene-BCIN_10g03050	377.4694395	991.7060953	-1.3936	1.63E-05	0.0020166	1167932	1169638	1502	Hypothetical protein
gene-BCIN_10g03900	2025.360589	4307.018527	-1.0885	0.00054627	0.024141	1495512	1497112	1476	Hypothetical protein
gene-BCIN_10g04080	26.30603474	74.42708205	-1.5004	0.001014	0.036019	1568181	1569921	1741	Hypothetical protein
gene-BCIN_10g04180	696.962997	10695.24423	-3.9397	2.74E-31	3.32E-27	1607542	1609267	1726	Hypothetical protein
gene-BCIN_10g04190	538.9497839	8968.857068	-4.0567	3.95E-29	2.39E-25	1610442	1613249	2808	Hypothetical protein
gene-BCIN_10g04490	177.2193466	425.2318727	-1.2627	0.00017572	0.011379	1724434	1726045	1612	Hypothetical protein
gene-BCIN_10g04690	423.6938491	873.115762	-1.0432	0.0013027	0.042312	1805585	1806966	1309	Bcfaf1
gene-BCIN_10g04710	18566.97668	42281.61564	-1.1873	0.0003094	0.016848	1808951	1811680	2639	Hypothetical protein
gene-BCIN_10g05070	193.1840262	576.4798971	-1.5773	2.10E-06	0.0004172	1916531	1917419	801	Hypothetical protein
gene-BCIN_10g05760	154.7784789	636.6722148	-2.0403	1.39E-09	8.48E-07	2198485	2201893	3215	Hypothetical protein

gene-BCIN_10g05840	217.949229	577.8699915	-1.4068	3.26E-05	0.0032127	2228289	2230580	2060	Putative MFS multidrug protein
gene-BCIN_10g05890	1542.181053	3208.599854	-1.057	0.00056969	0.024814	2262660	2264058	1140	Putative ribosome biogenesis protein rlp24 protein
gene-BCIN_10g05910	4505.710442	27652.37329	-2.6176	3.52E-13	4.26E-10	2272327	2276378	2954	Putative repetitive proline-rich cell wall protein
gene-BCIN_10g05950	5618.768301	12298.74849	-1.1302	0.00031331	0.016937	2282387	2285061	2463	Transcription regulator PAC1
gene-BCIN_11g00870	216.5854639	548.3637326	-1.3402	4.59E-05	0.0040594	310777	312018	1242	Hypothetical protein
gene-BCIN_11g00880	21.04794832	71.00988513	-1.7543	0.00026141	0.014931	312575	313527	953	Hypothetical protein
gene-BCIN_11g01020	574.8035239	1393.387734	-1.2775	0.00082296	0.031447	355043	356658	1520	Similar to fumarylacetoacetase
gene-BCIN_11g01050	2997.870999	15808.10907	-2.3987	1.37E-06	0.0002971	374740	376374	1586	Similar to homogentisate 1,2-dioxygenase
gene-BCIN_11g01590	523.1281572	1152.873397	-1.14	0.00035365	0.0187	517916	519321	1406	Hypothetical protein
gene-BCIN_11g01680	11062.01954	26798.49426	-1.2765	2.39E-05	0.0025835	540676	542910	2180	Hypothetical protein
gene-BCIN_11g01890	395.3751622	862.4856406	-1.1253	0.00049352	0.022829	614473	615584	1112	Hypothetical protein
gene-BCIN_11g02110	3717.755797	7998.659109	-1.1053	0.00029185	0.016137	701138	702876	1417	Bchmt1
gene-BCIN_11g02250	1373.055149	3815.314455	-1.4744	4.16E-06	0.0007007	763163	765008	1782	Bcnug1
gene-BCIN_11g02350	426.0034175	890.9188748	-1.0644	0.0008206	0.031447	791874	792811	872	Bcrrp1
gene-BCIN_11g02620	197.3736833	1089.954022	-2.4653	1.15E-05	0.0015174	902615	904565	1784	Similar to major facilitator superfamily transporter
gene-BCIN_11g02630	1867.375183	9249.447508	-2.3084	0.00027663	0.015585	906895	908137	1243	Hypothetical protein
gene-BCIN_11g02660	73.66680839	636.2172476	-3.1104	2.20E-05	0.0024783	915891	916873	983	Hypothetical protein
gene-BCIN_11g02670	392.9076916	3385.59734	-3.1071	3.88E-08	1.42E-05	918282	919449	1050	Hypothetical protein

gene-BCIN_11g02910	3487.244775	7251.87411	-1.0563	0.00066067	0.027304	997948	1002080	3770	Hypothetical protein
gene-BCIN_11g03040	3150.346975	7141.795606	-1.1808	0.00010245	0.0078019	1049315	1052534	2914	Hypothetical protein
gene-BCIN_11g03130	946.4843055	2178.642245	-1.2028	0.0001271	0.0090001	1081270	1082780	1458	Similar to ribosome biogenesis protein Ssf2
gene-BCIN_11g03140	1010.493321	2031.464566	-1.0075	0.0011564	0.039004	1082930	1084619	1643	Bcrrp3
gene-BCIN_11g03290	479.8413287	1159.907093	-1.2734	7.88E-05	0.0064483	1128287	1131544	3202	Hypothetical protein
gene-BCIN_11g03570	1172.757858	543.6780787	1.1091	0.00046306	0.021733	1249684	1251747	1950	Hypothetical protein
gene-BCIN_11g03580	1368.39126	4446.803965	-1.7003	0.0014264	0.044748	1258419	1261280	2535	Similar to ammonium transporter
gene-BCIN_11g03710	521.9011065	1577.184302	-1.5955	3.93E-07	0.0001057	1306374	1308699	1848	Hypothetical protein
gene-BCIN_11g04210	7238.680247	14445.69739	-0.99684	0.00071128	0.028564	1574932	1576271	1187	Similar to CORD and CS domain-containing protein
gene-BCIN_11g04250	659.3504273	1685.379491	-1.354	2.62E-05	0.0027829	1583451	1585487	1985	Hypothetical protein
gene-BCIN_11g04630	3587.702166	1844.119264	0.96013	0.0015608	0.047727	1703161	1705097	1653	Similar to glyoxylate reductase
gene-BCIN_11g05550	300.0827027	1052.303528	-1.8101	2.40E-08	9.67E-06	1973293	1975271	1821	Similar to cytochrome P450
gene-BCIN_11g05650	1305.25446	2683.327177	-1.0397	0.00074603	0.029619	2003596	2005217	1622	Similar to translation initiation protein Sua5
gene-BCIN_11g06150	175.6434649	45.28399588	1.9556	0.00048989	0.022816	2193924	2195020	1097	Hypothetical protein
gene-BCIN_11g06200	1156.102607	3285.919979	-1.507	1.63E-06	0.0003466	2211922	2213520	1599	Putative transcriptional regulator prz1 protein
gene-BCIN_11g06210	317.0897573	675.4962362	-1.0911	0.00074995	0.029677	2215096	2216205	1083	Hypothetical protein
gene-BCIN_11g06510	303.9291981	2799.267286	-3.2032	3.58E-14	7.22E-11	2304342	2305529	1135	Predicted protein
gene-BCIN_11g06520	494.2806114	3049.971016	-2.6254	4.67E-11	4.35E-08	2306184	2308590	1992	Pypothetical protein ZTR_07489

gene-BCIN_12g00190	1169.018331	3308.854948	-1.501	8.71E-06	0.0012065	82220	84126	1840	Bcgua1
gene-BCIN_12g00420	1553.886169	4175.117516	-1.4259	4.84E-06	0.0007814	139624	141709	2031	Bcnmd3
gene-BCIN_12g00630	423.9180407	1302.107258	-1.619	1.41E-05	0.0017957	221026	222214	1107	Hypothetical protein
gene-BCIN_12g00690	5927.72972	1935.3808	1.6149	0.00057439	0.02484	240371	255422	14888	Putative nonribosomal siderophore peptide synthase protein
gene-BCIN_12g01070	622.6586823	1380.787252	-1.149	0.00028816	0.016129	361978	364049	1916	Hypothetical protein
gene-BCIN_12g01530	63.98687558	478.196195	-2.9018	6.59E-07	0.00016	548753	551074	1892	Glycoside hydrolase family 16 protein
gene-BCIN_12g01540	33.34292706	320.0788034	-3.263	2.23E-05	0.0024783	552684	556830	3643	Hypothetical protein
gene-BCIN_12g01820	633.9493034	1449.930959	-1.1935	0.00086974	0.032606	633283	635860	2526	Putative ATP-dependent rna helicase dbp4 protein
gene-BCIN_12g02770	914.4228912	2136.018141	-1.224	8.39E-05	0.0066403	956256	957948	1304	Similar to thiamin pyrophosphokinase-related protein
gene-BCIN_12g03770	998.3555952	2230.028198	-1.1594	0.00023983	0.014237	1286621	1288133	1461	Bcnop53
gene-BCIN_12g04520	1014.700406	2430.753865	-1.2603	4.77E-05	0.0041853	1541211	1543101	1891	Hypothetical protein
gene-BCIN_12g04810	460.6715799	961.9934222	-1.0623	0.00083296	0.031718	1626380	1627762	1333	Hypothetical protein
gene-BCIN_12g04840	113.6639519	472.9442472	-2.0569	2.90E-06	0.0005496	1632957	1633918	757	Hypothetical protein
gene-BCIN_12g06600	483.617732	227.2341095	1.0897	0.000789	0.030919	2293491	2296086	2443	Similar to cytochrome P450 monooxygenase
gene-BCIN_12g06650	914.5671037	1890.841707	-1.0479	0.00079311	0.03098	2306040	2307178	1139	Bcrex4
gene-BCIN_13g00020	2982.819925	7428.617552	-1.3164	5.13E-05	0.0044343	14850	15783	934	Putative acyl-*N*-acyltransferase protein
gene-BCIN_13g00030	697.4595384	1607.723953	-1.2048	0.00011126	0.0082151	16302	17544	1243	Putative transcriptional protein
gene-BCIN_13g02570	638.5849579	267.7575319	1.254	0.00010756	0.0081072	880675	883460	2418	Putative hsp70 family protein

gene-BCIN_13g02780	3418.497753	6794.191216	-0.99094	0.0011727	0.039258	954394	956335	1780	Similar to MFS phospholipid transporter Git1
gene-BCIN_13g02980	419.6060773	890.4106235	-1.0854	0.00090051	0.033352	1041999	1043384	1268	Putative pre-rRNA-processing protein tsr2 protein
gene-BCIN_13g03020	756.1103294	1593.380698	-1.0754	0.00053142	0.023922	1063569	1065186	1506	Similar to branched chain alpha-keto acid dehydrogenase E1-beta subunit
gene-BCIN_13g03160	76.1767096	297.9216796	-1.9675	0.0011115	0.038236	1120786	1121643	805	Hypothetical protein
gene-BCIN_13g03450	2384.706214	1213.082476	0.97513	0.0014659	0.045749	1201744	1205806	4000	Bcmet10
gene-BCIN_13g03850	408.5959363	896.3166642	-1.1333	0.0014235	0.044748	1351313	1353987	2577	Hypothetical protein
gene-BCIN_13g03880	1234.654484	2840.02656	-1.2018	0.0001198	0.0086134	1360861	1362978	1865	Similar to adenylosuccinate lyase
gene-BCIN_13g04000	9664.101742	25775.53	-1.4153	0.00016589	0.01097	1412200	1414289	1681	Putative d-arabinitol 2-dehydrogenase protein
gene-BCIN_13g04700	699.4275714	2353.716375	-1.7507	8.00E-06	0.001148	1749578	1752166	1359	Putative flavin-nucleotide-binding protein
gene-BCIN_13g05380	208.2656636	93.33686577	1.1579	0.0010161	0.036019	2010326	2012064	1690	Hypothetical protein
gene-BCIN_13g05430	1230.31458	491.3675339	1.3242	4.25E-05	0.0038392	2066683	2067835	666	Hypothetical protein
gene-BCIN_13g05490	1125.567029	3020.841462	-1.4243	6.01E-06	0.0009334	2089752	2090585	834	Hypothetical protein
gene-BCIN_13g05800	33.68256406	114.833161	-1.7695	3.07E-05	0.0030927	2188964	2191785	2621	Similar to histidine acid phosphatase
gene-BCIN_13g05860	27.87684085	194.4299777	-2.8021	0.00038476	0.019694	2214065	2216050	1876	Putative glucose dehydrogenase protein
gene-BCIN_13g05870	36.10699812	269.571651	-2.9003	2.05E-13	2.75E-10	2216455	2218777	2201	Putative gmc oxidoreductase protein
gene-BCIN_14g00090	218.3152843	823.1065738	-1.9147	6.47E-09	2.90E-06	56740	58466	1444	Putative thermophilic desulfurizing enzyme family protein
gene-BCIN_14g00960	1141.728706	2592.976634	-1.1834	0.00015833	0.010711	428968	430689	1667	Similar to ATP-dependent RNA helicase has1
gene-BCIN_14g01090	353.0675507	1863.698	-2.4002	0.00018494	0.011664	501041	503949	2409	Similar to MFS sugar transporter

gene-BCIN_14g01190	625.0949111	1592.189954	-1.3489	0.000458	0.021597	535828	537727	1776	Similar to transcription factor Zn, C2H2
gene-BCIN_14g01410	557.2243157	2154.209531	-1.9508	1.68E-06	0.0003513	629204	631677	2229	Hypothetical protein
gene-BCIN_14g03600	185.855877	389.5760545	-1.0677	0.0015936	0.048122	1371045	1373299	1939	Hypothetical protein
gene-BCIN_14g03670	228.1700052	591.1760823	-1.3735	0.0010788	0.037431	1400541	1402865	2213	Putative tetrahydrofolylpolyglutamate synthase protein
gene-BCIN_14g03680	1507.410985	4057.953373	-1.4287	3.99E-06	0.0007	1403663	1406275	2350	Putative 4-coumarate-ligase 1 protein
gene-BCIN_14g03720	39.60724932	142.8995314	-1.8512	6.21E-06	0.0009512	1426743	1427522	695	Hypothetical protein
gene-BCIN_14g03730	254.3047161	608.1104284	-1.2578	0.00017842	0.011431	1428486	1430084	1546	Zinc finger C2H2-type protein
gene-BCIN_14g04470	675.9736692	1485.698926	-1.1361	0.00025851	0.014836	1752082	1754212	2052	Similar to amino acid transporter
gene-BCIN_14g04750	105.9986278	303.6667252	-1.5184	1.85E-05	0.0022191	1833746	1835236	1386	Hypothetical protein
gene-BCIN_14g05230	1623.547335	3275.124747	-1.0124	0.0011263	0.038595	2000562	2002302	1741	Bcutp5
gene-BCIN_14g05250	597.6076666	298.2061164	1.0029	0.0015763	0.047957	2008657	2012101	3302	Putative fad dependent oxidoreductase superfamily protein
gene-BCIN_14g05400	3640.639203	7684.518296	-1.0778	0.00041981	0.020762	2058772	2061213	2226	Hypothetical protein
gene-BCIN_15g00580	1933.998912	4151.792781	-1.1021	0.00025109	0.014617	225083	227972	2454	Similar to lipoxygenase
gene-BCIN_15g01030	1445.594119	578.4770999	1.3213	3.53E-05	0.0033686	389033	390376	1344	Carbohydrate esterase family 1 protein
gene-BCIN_15g01170	223.2344418	98.57656953	1.1792	0.0015812	0.047987	447848	451625	3147	Hypothetical protein
gene-BCIN_15g01260	492.3172413	1277.323481	-1.3755	4.30E-05	0.0038602	478252	479760	1509	Bcnob1
gene-BCIN_15g01360	1017.654581	480.0787371	1.0839	0.00067964	0.027803	511933	514095	1883	Putative gaba permease protein
gene-BCIN_15g01550	743.5787894	1786.088606	-1.2642	5.90E-05	0.0049972	564514	565896	1320	Similar to ribosome biogenesis protein

gene-BCIN_15g01620	710.6395077	1683.434741	-1.2442	0.00019229	0.01188	585357	587650	1947	Hypothetical protein
gene-BCIN_15g01990	138.7707576	333.2601299	-1.2639	0.00023384	0.014018	685843	686978	1055	Hypothetical protein
gene-BCIN_15g02240	804.3529066	1696.840352	-1.077	0.00044433	0.021462	772176	775157	2655	Putative benzoate 4-monooxygenase cytochrome P450 protein
gene-BCIN_15g02820	1193.257142	3518.061634	-1.5599	5.23E-07	0.0001319	973067	978115	4983	Hypothetical protein
gene-BCIN_15g03170	30.79475895	115.8028095	-1.9109	0.00044744	0.021462	1102800	1104637	1569	Similar to *O* -methyltransferase
gene-BCIN_15g03640	599.5202131	1313.221564	-1.1312	0.00038546	0.019694	1281152	1283510	1850	Hypothetical protein
gene-BCIN_15g03780	429.511371	965.3757804	-1.1684	0.00038216	0.019694	1330855	1333943	3089	Bcdhr2
gene-BCIN_15g03980	2.567574629	791.324628	-8.2677	4.14E-07	0.0001091	1393940	1395001	882	Hypothetical protein
gene-BCIN_15g03990	5.6480102	522.1652982	-6.5306	7.53E-13	8.29E-10	1397095	1398380	1134	Hypothetical protein
gene-BCIN_15g04000	7.722130403	62.25202644	-3.011	5.47E-06	0.0008598	1398385	1399653	1042	Putative Tat pathway signal sequence
gene-BCIN_15g04010	39.2704278	508.5527451	-3.6949	3.47E-19	1.05E-15	1400028	1401127	783	Hypothetical protein
gene-BCIN_15g04630	6772.369406	18522.40962	-1.4515	8.06E-06	0.001148	1578436	1580881	2206	Hypothetical protein
gene-BCIN_15g05370	2826.551821	5431.108643	-0.9422	0.0016304	0.048988	1881142	1884396	3031	Hypothetical protein
gene-BCIN_15g05400	48.82946235	154.2398901	-1.6594	3.18E-05	0.0031543	1892372	1893583	1162	Similar to ThiJ/PfpI family protein
gene-BCIN_15g05660	664.426815	2451.619131	-1.8836	0.00063827	0.026469	1978032	1980440	2233	Hypothetical protein
gene-BCIN_15g05690	185.1720319	19.14545545	3.2738	5.64E-14	9.75E-11	1993425	1994649	1132	Similar to oxidoreductase,short chain dehydrogenase
gene-BCIN_16g00970	656.085228	1479.991719	-1.1736	0.00015479	0.01065	408102	409961	1607	Hypothetical protein
gene-BCIN_16g02010	9852.97063	30195.94616	-1.6157	1.73E-07	5.36E-05	806484	807604	1121	Bcfes1

gene-BCIN_16g02330	754.7206377	1696.847733	-1.1688	0.00018976	0.011845	902685	903984	1243	Bcmak16
gene-BCIN_16g02580	67.49169795	199.2782978	-1.562	1.21E-05	0.0015711	1009360	1010707	1198	Putative sumo protein
gene-BCIN_16g02590	90.97553377	412.7951908	-2.1819	3.88E-10	3.13E-07	1011341	1014336	2742	Hypothetical protein
gene-BCIN_16g03140	3479.639813	6837.587497	-0.97455	0.0015452	0.04761	1198579	1200762	1893	Bccbf5
gene-BCIN_16g03570	1098.305298	373.1253815	1.5575	8.28E-05	0.0066068	1330116	1344559	14398	BcNRPS3, nonribosomal peptide synthetase
gene-BCIN_16g04370	386.7991908	833.9986991	-1.1085	0.00061884	0.026019	1597846	1600556	2663	Hypothetical protein
gene-BCIN_18g00010	6.907194175	96.78888395	-3.8087	5.35E-09	2.49E-06	2641	4925	2285	Putative heterokaryon incompatibility protein
gene-BCIN_18g00140	43.45045735	157.635404	-1.8591	4.61E-06	0.0007549	159943	162649	2467	Putative C2H2 finger domain-containing protein

Table S4 Genes and primers used for qRT-PCR

Gene ID	Primer	Sequence (5'-3')	Amplicon length	Temperature of annealing	Temperature of melting	Amplification efficiency(%)	Relevant characteristics
Bcin09g04490	Glycoside 65-F	AATGCTTGCCCGTAACTTCC	229bp	58.8	87.01	103.162	Bcin09g04490 for qPCR
	Glycoside 65-R	TCGTCTGTTGTTTCGTTGCC		59.3			
Bcin01g04230	Bcmet16-F	TGCAAAGAGAGAGAGCTTGGAC	231bp	60.3	83.95	98.891	Bcin01g04230 for qPCR
	Bcmet16-R	TTCTCAGTGCCCTTCCATCTTC		60.0			
Bcin05g02490	Bcmxr1-F	ATGCCCAATTTCCTCAACCG	184bp	58.8	85.78	99.493	Bcin05g02490 for qPCR
	Bcmxr1-R	CGTCGTACAAGCCTTTTCCG		59.6			
Bcin07g01300	BcCHSVII-F	CCGTCGTTTCAAGATTTGGC	228bp	58.0	84.86	102.645	Bcin07g01300 for qPCR
	BcCHSVII-R	GCAGCGGGTTTGAATGTTTC		58.6			
Bcin03g06050	Bcfap7-F	GTGGATTCGACGCTGTTGTATG	170bp	60.0	80.81	106.563	Bcin03g06050 for qPCR
	Bcfap7-R	TCCACCTCGTCACTTGTATCAC		59.8			
Bcin11g02110	Bchmt1-F	ACCGTCGAGATTAAGGCTGTC	205bp	59.9	83.87	96.785	Bcin11g02110 for qPCR
	Bchmt1-R	TGTGAGGACCAGTGCTGAATC		60.0			
Bcin01g06930	BccarA-F	TGTTGGTGTGGTTGTTGCAG	222bp	59.5	83.51	99.001	Bcin01g06930 for qPCR
	BccarA-R	TGCTCAGGATCAACGAAAGC		58.6			
Bcin01g00110	BcBOA11-F	TCGCCGCAGTAACATTGAAG	241bp	58.9	82.24	95.352	Bcin01g00110 for qPCR
	BcBOA11-R	ACTCGGAAACCTTGCCTTTC		58.4			
Bcin14g03730	Zinc C2H2-F	AGCCCAACCGAAAACCTTTC	186bp	59.0	83.23	101.131	Bcin14g03730 for qPCR
	Zinc C2H2-R	ACTTCCCGCTCTCTAGATGTTG		59.6			
Bcin16g02010	Bcfes1-F	TGCCATGAATGTGCTCTTTGG	158bp	59.5	83.14	93.241	Bcin16g02010 for qPCR
	Bcfes1-R	TGTTGGCATTGTCGAGGTTC		58.8			
Bcin12g00420	Bcnmd3-F	GCGAGTTTTATCTGGACGGAAC	178bp	59.7	81.87	106.542	Bcin12g00420 for qPCR
	Bcnmd3-R	CACAAGCTCTCCATGTATTCGC		59.7			
Bcin15g01260	Bcnob1-F	ATCCGCCAGCTCAAAACTTG	234bp	59.1	83.71	94.236	Bcin15g01260 for qPCR
	Bcnob1-R	ATTCTTTCCGTTCGCTGTCC		58.6			
Bcin01g10130	Bcdug2-F	TGGACCAGGTTTTTCGGAAG	274bp	58.0	85.13	98.352	Bcin01g10130 for qPCR
	Bcdug2-R	ACATCCGGCAACTGAAGTTC		58.5			
Bcin01g10150	Bcpsd-F	GTTCGATTCGCCAAGGAGATC	186bp	59.1	82.93	105.427	Bcin01g10150 for qPCR
	Bcpsd-R	TTGCCCGAAAATCCTACGTG		58.6			

Bcin02g03350	Bccox17-F	TGGCGAAGTTTCTGCAAAGC	154bp	60.0	83.66	108.342	Bcin02g03350 for qPCR
	Bccox17-R	CCCAAAGCCAGCCATACAAC		59.5			
Bcin16g03570	BcNPRS-F	GCGAACAAGAAAGTGGCAAC	192bp	58.5	83.34	97.452	Bcin16g03570 for qPCR
	BcNPRS-R	ATTGCTCCGAACCCAGAATG		58.2			
Bcactin	Bcactin-F	CTCTATTCAAGCCGTCCTCTCC	162bp	59.4	83.69	93.461	Reference gene for qPCR
	Bcactin-R	TAATCAGTCAAATCACGACCAGC		57.4			
